# Marek’s disease virus-1 unique gene LORF1 is involved in viral replication and MDV-1/Md5-induced atrophy of the bursa of Fabricius

**DOI:** 10.1371/journal.ppat.1012891

**Published:** 2025-02-03

**Authors:** Chenyi Bao, Jun Chu, Qi Gao, Shasha Yang, Xiaoyu Gao, Wenwen Chen, Fuchun Yang, Fei Jiang, Chenxi Tong, Mingyi Lei, Linlin Jiao, Jitong Li, Kexin Wei, Xue Lian, Kai Li, Suresh Kumar Tikoo, Nikolaus Osterrieder, Lorne A. Babiuk, Yufeng Li, Yong-Sam Jung, Yingjuan Qian

**Affiliations:** 1 The Sanya Institute of Nanjing Agricultural University, Laboratory of Emerging Animal Diseases and One Health, Nanjing Agricultural University, Nanjing, China; 2 MOE Joint International Research Laboratory of Animal Health and Food Safety, College of Veterinary Medicine, Nanjing Agricultural University, Nanjing, China; 3 Jiangsu Key Laboratory for High-Tech Research and Development of Veterinary Biopharmaceuticals, Jiangsu Agri-Animal Husbandry Vocational College, Veterinary Bio-Pharmaceutical, Taizhou, China; 4 Institute of Poultry Science, Shandong Academy of Agricultural Sciences/Shandong Provincial Key Laboratory of Poultry Diseases Diagnosis and Immunology, Jinan, China; 5 School of Animal Husbandry and Veterinary Medicine, Jiangsu Vocational College of Agriculture and Forestry, Jurong, China; 6 Institute of Urban Agriculture, Chinese Academy of Agricultural Sciences, Chengdu, China; 7 Vaccine and Infectious Disease Organization-International Vaccine Center (VIDO-InterVac), University of Saskatchewan, Saskatoon, Canada; 8 Tierärztliche Hochschule Hannover, Hannover, Germany; 9 Institut für Virologie, Freie Universität Berlin, Berlin, Germany; 10 Faculty of Agricultural, Life and Environmental Science, University of Alberta, Edmonton, Canada; Leibniz Institute of Virology (LIV), GERMANY

## Abstract

Marek’s disease virus (MDV), an alphaherpesvirus, causes severe immunosuppression and T cell lymphomas in chickens, known as Marek’s disease (MD), an economically important poultry disease primarily controlled by vaccination. Importantly, it also serves as a comparative model for studying herpesvirus-induced tumor formation in humans. MDV encodes more than 100 genes, most of which have unknown functions. MDV LORF1 is unique to serotype I MDV (MDV-1), lacking homologs in other herpesviruses, and has not been explored yet. To this end, an infectious bacterial artificial chromosome (BAC) harboring the complete genome of the MDV-1 very virulent strain Md5 was generated, and the rescued rMd5 maintained biological properties similar to the parental virus both *in vitro* and *in vivo*. Subsequently, rMd5ΔLORF1, a recombinant Md5 virus deficient in pLORF1 expression, was generated by a frameshift mutation in the LORF1 gene. Chickens infected with rMd5ΔLORF1 exhibited a lower mortality rate and delayed bursal atrophy than those infected with the parental rMd5 and the revertant virus (rMd5-reLORF1). Consistently, viral loads of rMd5ΔLORF1 were obviously lower than those of rMd5 or rMd5-reLORF1 in the bursa, but not in the spleen. Importantly, we found that pLORF1 deficiency impairs viral replication in bursal B cells. Furthermore, we showed that pLORF1 associated with the cellular membrane, interacted with MDV structural proteins, and exhibited punctate colocalization with tegument or capsid proteins in the cytoplasm. Taken together, this study demonstrates for the first time that the MDV-1 unique gene LORF1 is involved in MDV-induced bursal atrophy but not in tumor formation.

## Author summary

Marek’s disease virus (MDV) is an extensively studied alphaherpesvirus since it is not only a causative agent of Marek’s disease (MD), a highly contagious poultry neoplastic disease posing a significant economic impact on the poultry industry worldwide, but also a comparative model for studying human oncogenic viruses. Although MD has been effectively controlled by massive vaccination since 1969, the continuous emergence of higher virulent strains creates challenges for preventing future outbreaks due to vaccine failure. Therefore, the characterization of unknown genes related to MDV-induced tumor formation and/or immune suppression is of great importance. In this study, we showed for the first time that LORF1, a unique gene of serotype 1 MDV, appears to affect viral replication within the bursa, consequently influencing bursal atrophy. Our findings reveal an essential role of LORF1 in immune suppression, suggesting its potential as a target gene for the development of an MDV gene deletion vaccine.

## Introduction

Marek’s disease virus (MDV) is a highly pathogenic, immunosuppressive, and oncogenic alphaherpesvirus primarily affecting chickens [1]. MDV belongs to the genus *Mardivirus*, subfamily *Alphaherpesvirinae*, and family *Herpesviridae*. The genus *Mardivirus* comprises three closely related but distinct species: MDV-1 (*Gallid* herpesvirus 2, GaHV-2), GaHV-3 (previously MDV-2), and turkey herpesvirus 1 (HVT; *Meleagrid* herpesvirus 1, MeHV-1; previously MDV-3). The sequence similarity of viral proteins of the three species ranges from 50% to 80% [2–4]. However, only MDV-1 causes Marek’s disease (MD) in chickens, which is not only economically important to the global poultry industry but also an excellent natural T cell lymphoma model to improve our understanding of herpesvirus-induced tumorigenesis in humans [5]. For example, MDV-induced lymphomas share many similarities with lymphoid tumors associated with human herpesviruses, such as Epstein-Barr virus (EBV) [6]. Despite the success of vaccines in reducing losses from MD over the past four decades, the virus has evolved to be more virulent in vaccinated chickens, posing a threat to the effectiveness of the vaccine [7]. Three different pathotypes of the virus have been identified based on their vaccine escape abilities: virulent (v), very virulent (vv), and very virulent plus (vv+) [8]. A better understanding of the pathogenicity and oncogenicity of MDV is essential not only to clarify virus-associated pathogenesis but also to develop more effective vaccines to combat the infection.

Bacterial artificial chromosome (BAC) clones allow rapid manipulation of viral genomes for characterizing gene function and generating recombinant vaccines. In a seminal study, the complete genome of an attenuated strain of MDV vv+ 584A virus (584Ap80C) was established as a BAC clone [9]. The BAC sequence was inserted into the US2 locus of the MDV genome, and the recombinant virus was enriched with a selection media containing mycophenolic acid, xanthine, and hypoxanthine [9]. BAC clones from MDV pathogenic and vaccine strains were later extensively developed following the same principle [10–18]. Since the beginning of the 21st century, multiple key genes involved in MDV-induced pathogenesis, tumorigenesis, and immune suppression have been identified through genetically specific deletion and insertion modifications to MDV genes using these MDV BAC clones [19–27]. Meanwhile, recombinant MDV vaccine candidates deficient in important virulent genes, such as Meq, have been explored and shown vaccine potential [28,29]. MDV-1 encodes at least 100 gene products, with the majority situated in the unique long (U_L_) and unique short (U_S_) regions of the viral genome [30]. These genes exhibit homology to those found in herpes simplex virus (HSV-1) and are essential for various viral processes such as genomic DNA replication, gene expression, virus assembly, and morphogenesis [5,31]. For instance, genes like glycoprotein B (gB) and gM in the U_L_ region are critical for MDV replication *in vitro* [9,32]. UL28 and UL33 are crucial for MDV packaging and maturation [33]. Genes in the U_S_ region encode important viral envelope glycoproteins (gD, gI, and gE), and the US3 protein kinase is involved in DNA replication and gene expression [34–38]. Several unique genes, such as Meq, vIL8, vTR, pp38, and vLIP, identified within the repeat long (R_L_) regions and the U_L_ region, are known to play significant roles in MDV pathogenesis and/or oncogenesis [19,21,39–46]. However, many other open reading frames (ORFs) unique to MDV or to *Mardivirus* viruses in general have not been thoroughly studied. Notably, there are 16 unique ORFs in the R_L_ region, 12 in the U_L_ region, and 4 in the U_S_ region [31,47]. These unique genes, absent in GaHV-3, HVT, or other herpesviruses, likely contribute to the distinct biological properties of these viruses. Despite this, many of these ORFs remain uncharacterized, with limited homology to known genes or sequence motifs. Some ORFs overlap with other MDV genes, and mutations affecting start or stop codons can help elucidate their specific functions without altering the protein sequence of the overlapping genes. This necessitates a technological platform for precise manipulation of the viral genome to better elucidate their functions.

LORF1, the first ORF of the U_L_ region of the MDV-1 genome, was first identified as ORF-2, encoding a putative protein consisting of 333 amino acids [31,48], or MDV009 due to its presence in MDV-1, but not in MDV-2 or HVT [30]. Two unique peptides of pLORF1 were identified through proteomic analysis of Md5-infected chicken embryo fibroblasts (CEFs) cells [49]. Expression of LORF1 mRNA was detected in an MDV-transformed tumor cell line [50]. However, its role in MDV pathogenesis remains unknown [47]. In this study, we demonstrate that pLORF1 is involved in MDV-1-induced immune suppression but not in tumor formation.

## Results

### Construction of a BAC containing the complete MDV-1 genome

**Fig 1:**
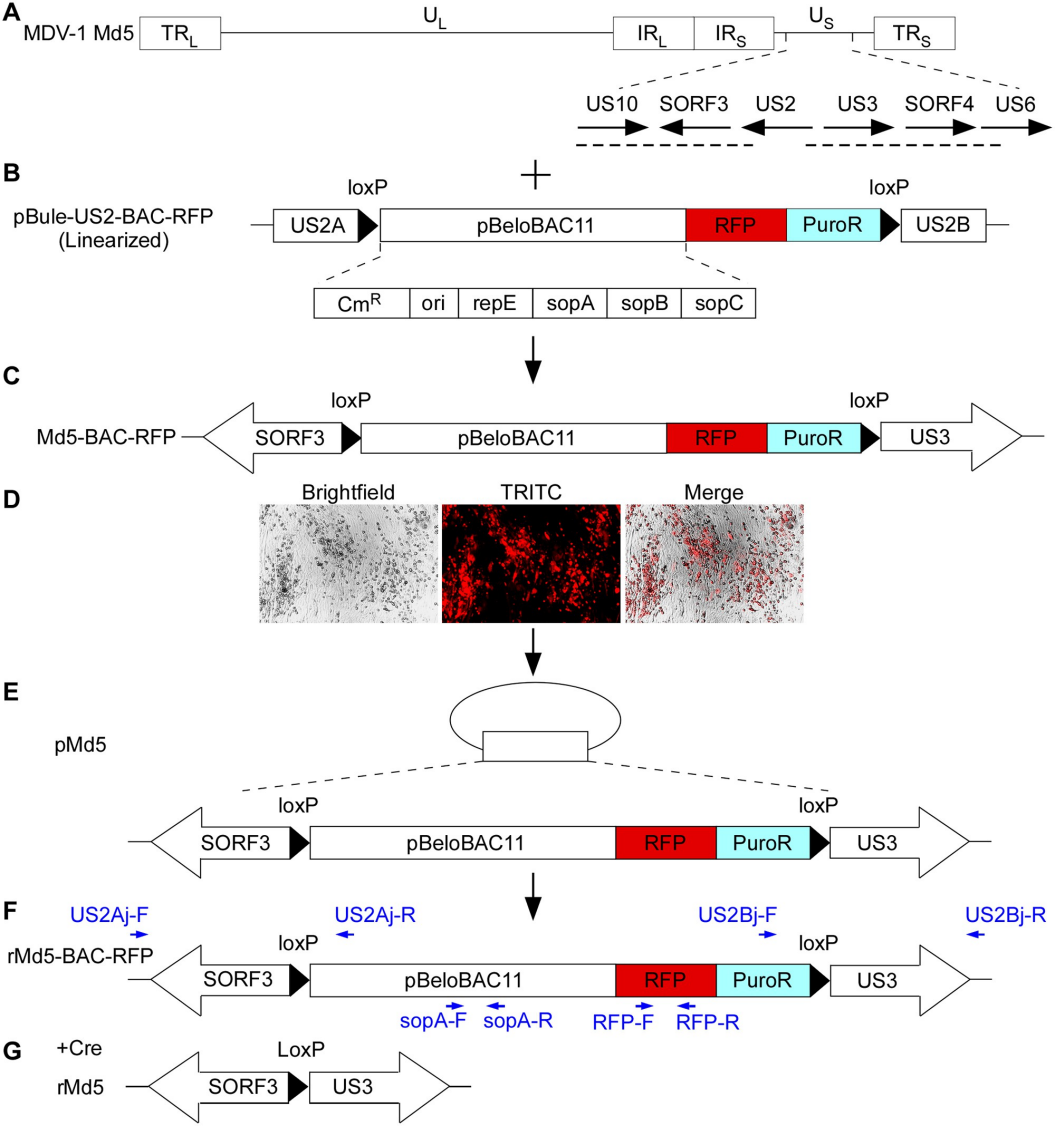
Strategies for constructing the MDV-1 Md5 BAC clone. (A) Schematic of the linear MDV-1 Md5 genome and a portion of the unique short region (U_S_) containing US10 to US6 ORFs and the location of homologous arms (horizontal dashed line). (B) A linearized transfer vector, pBlue-US2-BAC-RFP, includes homologous arms (US2A and US2B), a loxP-flanked partial sequence of the pBeloBAC11 plasmid, and a selection marker expression cassette (RFP and puromycin). (C) CEFs were transfected with linearized pBlue-US2-BAC-RFP, followed by MDV-1 Md5 virus infection. Through classical homologous recombination, the transfer vector was introduced into the MDV-1 genome between SORF3 and US3 to yield a recombinant MDV-1, Md5-BAC-RFP. (D) Microscopic observation of red fluorescent plaques of Md5-BAC-RFP obtained by plaque purification in CEFs. (E) Md5-BAC-RFP viral DNA was isolated from infected CEFs and electroporated into *E*. *coli* DH10B cells to generate infectious pMd5 clones. (F and G) CEFs were transfected with pMd5 DNA with or without the Cre recombinase expression plasmid and produced recombinant virus rMd5-BAC-RFP (F) or rMd5 (G). The blue arrows indicate the locations of primers used to confirm the removal of the BAC sequence.

**Fig 2:**
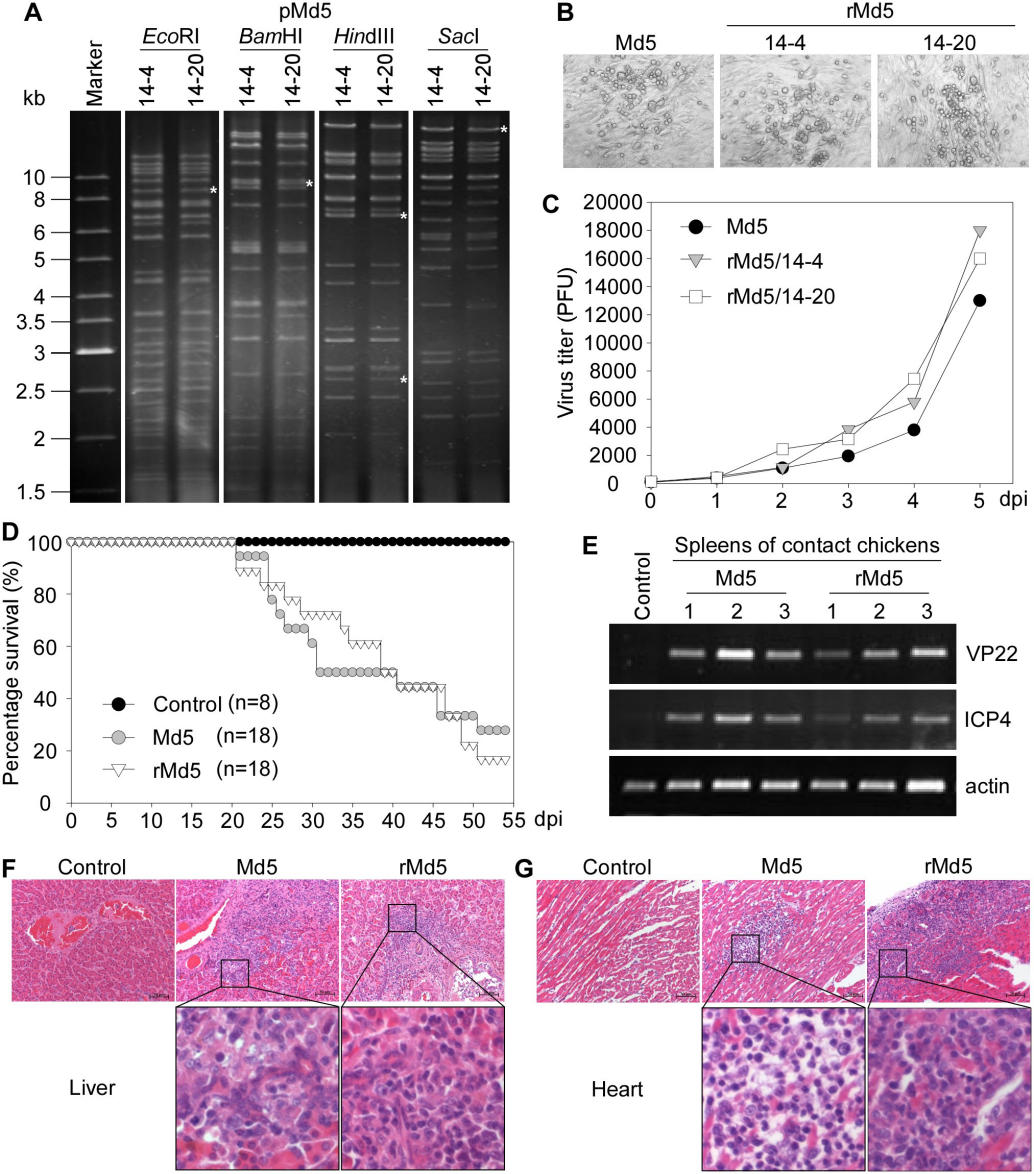
Characterization of the BAC-derived viruses *in vitro* and *in vivo*. (A) RFLP analysis of BAC DNA. Electrophoresis of pMd5 DNAs (clones 14–4 and 14–20) that were cleaved with *Eco*RI, *Bam*HI, *Hin*dIII, or *Sac*I endonucleases. The bands that indicate the insertion of the transfer vector were marked with asterisks. (B) The microscopic images of viral plaques of Md5, rMd5/14-4, and rMd5/14-20 in CEFs at 5 dpi. (C) The growth curve of Md5, rMd5/14-4, or rMd5/14-20 was performed as described in “Materials and Methods.” Each value represents the mean of two independent duplicates. (D-G) One-day-old SPF chickens were randomly assigned to receive intraperitoneal injections of either Md5 or rMd5 at a dose of 1,000 PFU, or DMEM as a control. The chickens were kept in isolators for 54 days together with contact chickens housed in the same isolators as either the Md5 or rMd5 group. (D) Survival curves of chickens inoculated with the indicated viruses and the control group. (E) The viral load in contacted chickens. The levels of the MDV VP22 and ICP4 genes were measured by PCR using DNA purified from the spleen tissues of mock-treated or contact chickens from the Md5 or rMd5 groups at 54 dpi. (F and G) Liver (F) and heart (G) tissues from chickens at 42 dpi in each group were stained with H&E. The black boxes indicate large populations of lymphoid tumor cells scattered in the Md5- or rMd5-infected tissues. Scale bar: 50 μm.

In order to explore the role of unknown genes of MDV-1, an MDV-BAC clone containing the full-length MDV-1 Md5 genome was generated, as illustrated in Fig 1. The transfer vector pBlue-US2-BAC-RFP carries a homologous recombination cassette consisting of homology arms (US2A and US2B), a loxP-flanked partial sequence of the pBeloBAC11 plasmid, and a selection marker expression cassette (RFP and puromycin) (Fig 1B). The pBeloBAC11 contains the Cm^R^ gene, the Ori2 replicon, and the replication and partition genes (repE, sopA, sopB, and sopC). This transfer vector directs the insertion of BAC elements into the US2 gene of MDV (Fig 1A) as previously reported [9]. CEFs were transfected with the linear pBlue-US2-BAC-RFP vector and subsequently infected with the Md5 virus. Instead of using selection medium to enrich recombinant viruses, a common method used in constructing MDV-BAC [9–18], we utilized the red fluorescent protein (RFP) to visualize recombinant virus-formed plaques for purification. Transfected/infected cells were trypsinized and serially diluted onto fresh CEFs in 96-well plates. Wells containing a single RFP-positive plaque were selected to repeat the above steps. After six rounds of plaque purification, the propagation of the recombinant Md5-BAC-RFP virus (Fig 1C) was observed with the aid of a fluorescence microscope, and the images revealed that all plaques fluoresced in red (Fig 1D). The circular viral DNAs (Fig 1E) from these cultures were transformed into *E*. *coli* DH10B using electroporation. Chloramphenicol-resistant colonies were screened using restriction fragment length polymorphism (RFLP) analysis. Two independently derived BAC clones, named pMd5/14-4 and pMd5/14-20, were confirmed by restriction enzyme digestion using *Eco*RI, *Bam*HI, *Hin*dIII, and *Sac*I, respectively. No noticeable deletions, insertions, or rearrangements in restriction patterns were identified in the pMd5 DNA by comparing electrophoretic profiles with those predicted by the software (Fig 2A and S1A Fig). The asterisks indicate the fragments generated due to the insertion of the transfer vector into the Md5 genome.

To rescue the recombinant rMd5-BAC-RFP virus (Fig 1F) from pMd5 BAC (Fig 1E), CEFs were transfected with pMd5/14-4 or pMd5/14-20 DNA, and red fluorescent plaques were visualized at 5 days post-transfection (S1B Fig). To remove the BAC vector flanked by loxP sites from the pMd5 genome to obtain a minimally modified MDV, rMd5 (Fig 1G), CEFs were co-transfected with pMd5 DNA and a Cre expression plasmid. Non-fluorescent BAC-excised rMd5 viruses were plaque-purified and confirmed by PCR amplification of the DNA from rMd5-infected CEFs with primers shown in Fig 1F (blue arrows). The results showed that the US2 gene junctions, RFP, and sopA were exclusively detected in rMd5-BAC-RFP, but not in rMd5 samples, while the VP22 remained intact in all viruses (S1C Fig). The recombinant viruses, rMd5/14-4 and rMd5/14-20, induced typical MDV cytopathic effects, similar to those of the wild-type Md5 virus (Fig 2B). In addition, the growth kinetics of rMd5/14-4 and rMd5/14-20 closely resembled that of the parental Md5 on CEFs (Fig 2C). Since both rMd5/14-4 and rMd5/14-20 displayed indistinguishable properties *in vitro*, clone 14–20 was used for further experiments.

### Characterization of BAC-derived MDV-1 *in vivo*

The virulence of rMd5 was determined in one-day-old SPF chickens over a 54-day period, with Md5 as a control. MDV infection caused clinical symptoms or death, which were monitored and showed that chickens started to die from 21 dpi, and the median survival time was 41 dpi in both rMd5- and Md5-infected groups (Fig 2D). In addition, to examine the horizontal transmission of rMd5, the levels of VP22 and ICP4 genes in the spleens of contact chickens were analyzed by PCR at the end of the experiment. The results showed that VP22 and ICP4 were detected in all contact chickens in both Md5 and rMd5-infected groups, indicating that the rMd5 virus maintains the ability to spread horizontally from infected chickens to contact chickens (Fig 2E).

Since MDV infection induces immunosuppression and causes abnormalities in lymphoid organs in chickens, chicken bursas and spleens were examined at 28 dpi, and those organs showed that both rMd5 and Md5 infections induced bursal atrophy and spleen enlargement (S1D Fig). In addition, MDV-induced lymphoma formation was also observed in the livers of both infection groups at 42 dpi (S1E Fig). Moreover, histological examination of dissected liver and heart tissues showed typical lymphocyte infiltration in these tissues of both Md5 and rMd5-infected chickens (Fig 2F and 2G, and S1F Fig), as well as in liver tissues of contact chickens (S1G Fig). These results suggest that the BAC-derived rMd5 virus maintains the ability to induce MD in chickens, comparable to the parental Md5 virus.

### Construction and characterization of recombinant Md5 viruses *in vitro*

**Fig 3:**
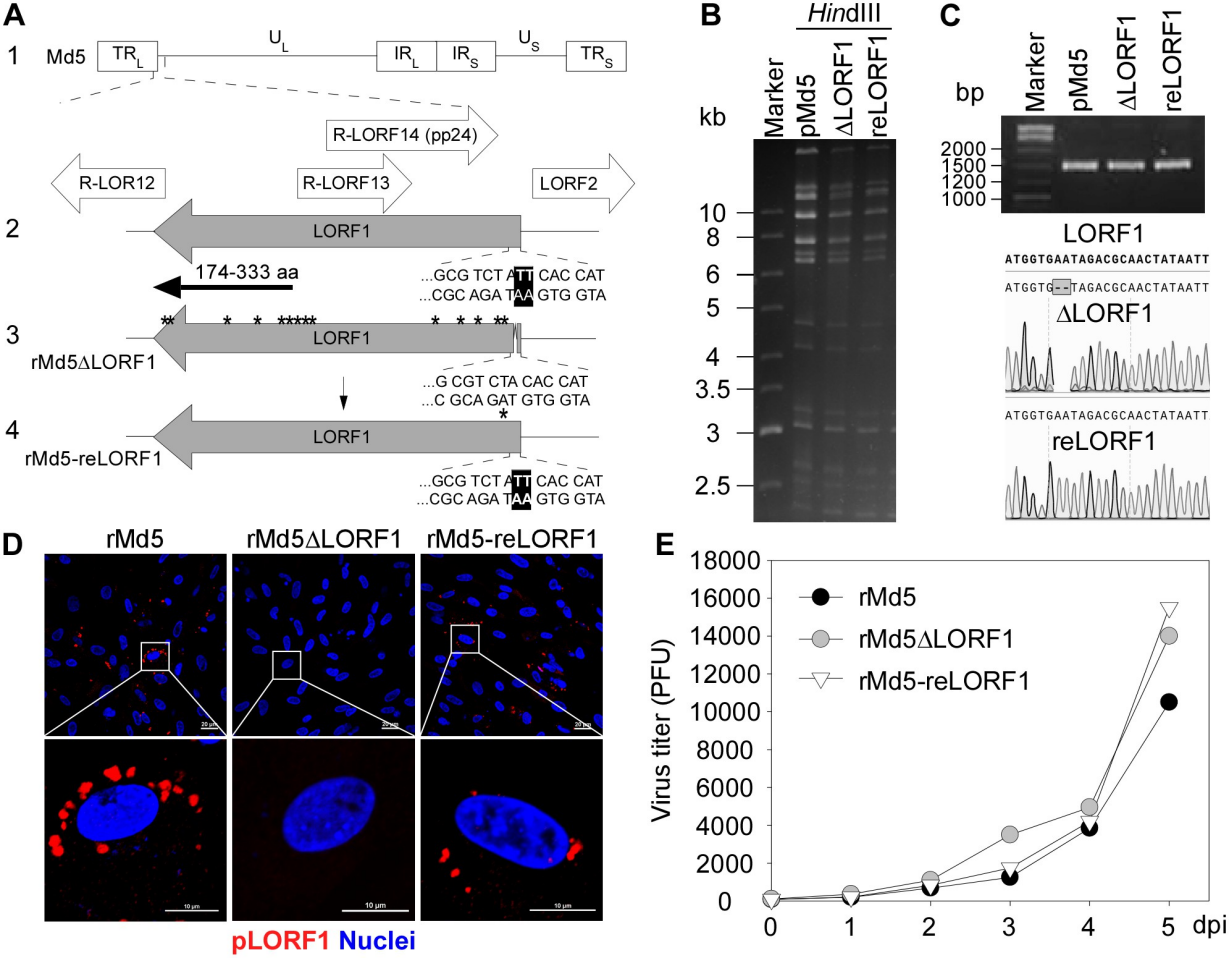
Construction of the pLORF1-deficient virus. (A) Schematic diagrams of the genome structure of MDV Md5 and the mutant viruses. Line 1: the wild-type MDV Md5 genome; Line 2: a segment of the Md5 genome that encompasses the R-LORF12 to LORF2 ORFs; Line 3: the rMd5ΔLORF1 virus with newly created stop codons (asterisks) due to a 2-bp deletion in the LORF1 gene; Line 4: the rMd5-reLORF1 virus with restored wild-type LORF1 ORF by re-introduction of 2 bp in the mutant rMd5ΔLORF1. The C-terminal region of pLORF1 (174–333 aa) was expressed as an antigen to produce anti-pLORF1 antibodies (black arrow). (B) RFLP analysis of *Hin*dIII-digested BAC DNAs from pMd5, pMd5ΔLORF1, or pMd5-reLORF1. (C) PCR amplification and sequencing results of the LORF1 gene in pMd5ΔLORF1 or -reLORF1. (D) The subcellular localization of pLORF1. CEFs were infected with rMd5, rMd5ΔLORF1, or rMd5-reLORF1 for 3 days, followed by fixation and staining with anti-pLORF1 antibody and Alexa 555-conjugated goat anti-mouse IgG antibody (red). The nuclei were stained with DAPI (blue). Original magnification: ×600. (E) The growth curve of rMd5, rMd5ΔLORF1, or rMd5-reLORF1. Each value represents the mean of two independent duplicates.

MDV LORF1 is unique to MDV-1 and has no homolog in other herpesviruses [30]. To date, its function in MDV pathogenesis remains unknown [47]. According to the MDV genome structure, the LORF1 gene completely overlaps with R-LORF14 and R-LORF13, partially overlaps with R-LORF12, and is adjacent to LORF2 (Fig 3A). Therefore, rather than creating a full-length deletion, a LORF1 frameshift mutant, rMd5ΔLORF1, was constructed by deleting AA at base positions 7 and 8 using the Red-mediated recombination system [51], which generated in-frame 14 premature stop codons, among which one locates at the second position after the translational start site (Fig 3A). Meanwhile, a revertant virus, rMd5-reLORF1, was also generated using Md5-BAC (Fig 3A). The RFLP analysis showed that both pMd5ΔLORF1 and pMd5-reLORF1 displayed the expected *Hin*dIII-digested DNA profiles (Fig 3B). Since the 2-bp deletion in the LORF1 gene cannot be differentiated only based on DNA fragment size, PCR products targeting the deletion region of pMd5ΔLORF1 and pMd5-reLORF1 were sequenced and showed that the LORF1 gene was precisely edited in each clone (Fig 3C). rMd5ΔLORF1 and rMd5-reLORF1 were rescued as above. To confirm the effect of the mutation on LORF1 expression, the C-terminal region of pLORF1 (174–333 amino acids, black arrow in Fig 3A) was expressed as an antigen to produce antisera against pLORF1 in mice. Confocal imaging revealed that the anti-pLORF1 antibody detected punctate localization of pLORF1 in the cytoplasm of cells infected with rMd5 and rMd5-reLORF1, which was absent in cells infected with rMd5ΔLORF1 (Fig 3D). Finally, the growth curve of the rMd5ΔLORF1 virus was comparable to the parental and revertant viruses (Fig 3E), suggesting that silencing the expression of the LORF1 gene had no effect on Md5 replication in CEFs.

Previously, studies demonstrated that the deletion of Meq completely disabled Md5-induced tumor formation [39]. Therefore, a full-length Meq-deleted virus, rMd5ΔMeq, was simultaneously constructed as a control (S2A and S2B Fig) and confirmed by RFLP analysis and PCR amplification (S2C and S2D Fig). Consistent with previous findings, the deletion of Meq did not affect Md5 replication in CEFs (S2E Fig).

### Characterization of recombinant Md5 viruses *in vivo*

**Fig 4:**
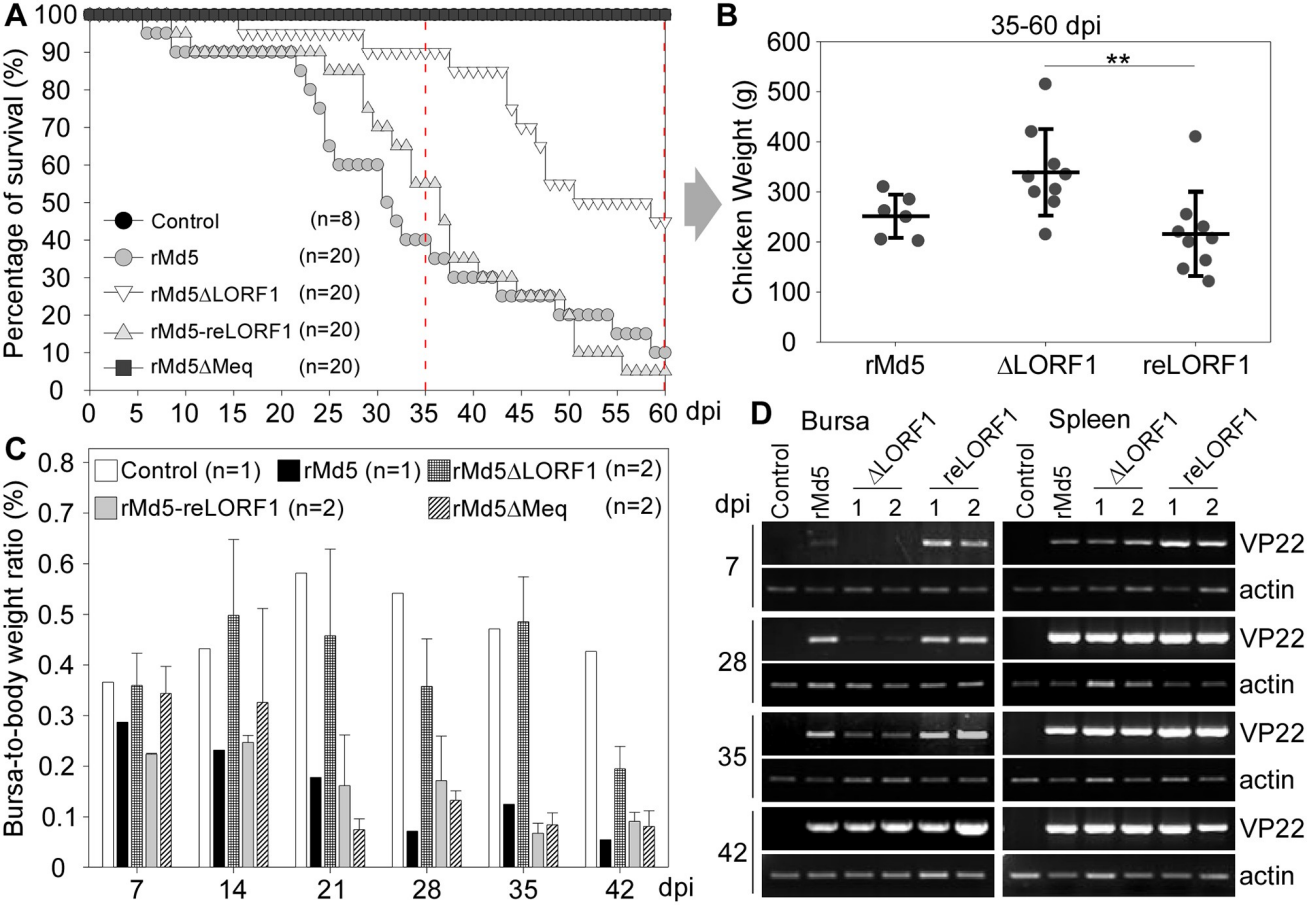
Loss of pLORF1 attenuates MDV-induced pathogenicity and bursal atrophy. The animal experiment was performed as described in “Materials and Methods.” (A) Survival curves of chickens inoculated with the indicated viruses and the control group. The time interval indicated by the two red dotted lines represents when 82% (9/11 chickens) of rMd5ΔLORF1-infected chickens died. (B) The body weight of dead chickens in each group from 35 dpi to 60 dpi. (C) The bursa to body weight ratio of chickens from each group at the indicated time points. Results represent the mean value, with error bars indicating the standard error of the mean. (D) The level of the MDV VP22 gene was measured by PCR using DNA purified from bursa and spleen samples of chickens from each group at the indicated time points.

To investigate the role of pLORF1 in MDV pathogenesis *in vivo*, one-day-old SPF chickens were intraperitoneally inoculated with 1,000 PFU of the parental virus (rMd5), the mutant virus (rMd5ΔLORF1), the revertant virus (rMd5-reLORF1), or the Meq-deleted virus (rMd5ΔMeq), respectively, along with DMEM as a negative control. The MD symptoms and survival of infected chickens were monitored for 60 days. Compared to the rMd5 and rMd5-reLORF1 groups, the rMd5ΔLORF1 group showed delayed progression of MD and lower mortality rates. The mortality rate of the rMd5ΔLORF1 group was 55% at the end of the experiment, while it was 90% for the rMd5 group and 95% for the rMd5-reLORF1 group (Fig 4A). There were no MDV-associated deaths in the rMd5ΔMeq group (Fig 4A), as previously reported [39]. In addition, 82% (9/11 chickens) of the chickens that died due to rMd5ΔLORF1 infection were clustered between 35 and 60 dpi (Fig 4A, marked by two red dotted lines). Interestingly, we found that rMd5ΔLORF1-infected chickens that died during this period had significantly higher body weights than those in the rMd5 and rMd5-reLORF1 groups (Fig 4B). Meanwhile, bursal atrophy in rMd5ΔLORF1-infected chickens was not as severe as that in the case of the rMd5 and rMd5-reLORF1 viruses (S3A Fig).

To confirm the aforementioned findings, another flock of one-day-old chickens was subjected to the same treatments (DMEM, rMd5, rMd5ΔLORF1, rMd5-reLORF1, and rMd5ΔMeq) and randomly selected for euthanasia at 7, 14, 21, 28, 35, or 42 dpi, respectively. During the observation period, chickens from the rMd5ΔLORF1 group exhibited progressively higher body weights compared to those in the rMd5, rMd5-reLORF1, and rMd5ΔMeq groups until 35 dpi (S3B Fig and S1 Table). To evaluate the effect of Md5-induced bursal atrophy, bursas were dissected, photographed (S3C Fig), and weighed (S1 Table). To rule out the effect of absolute weight of body or bursa due to exceptional individuals [52], the bursa/body weight ratio was calculated and shown to be continuously higher in chickens infected with rMd5ΔLORF1 since 14 dpi than in the rMd5-reLORF1 and rMd5ΔMeq groups (Fig 4C). The viral loads in the bursa and spleen tissues were analyzed by measuring the level of genomic VP22 and showed that it was substantially lower in the rMd5ΔLORF1-infected bursa samples than in rMd5- or rMd5-reLORF1-infected samples at 7, 28, and 35 dpi, but not at 42 dpi (Fig 4D), while it remained unchanged in the spleen (Fig 4D). To examine the tumorigenesis of recombinant viruses, histological assessment of liver tissues showed that liver lymphocyte infiltration in rMd5ΔLORF1-infected chickens was as severe as in the rMd5 and rMd5-reLORF1 groups at 35 dpi (S4 Fig).

### Histopathological analysis of bursal tissues infected by recombinant viruses

**Fig 5:**
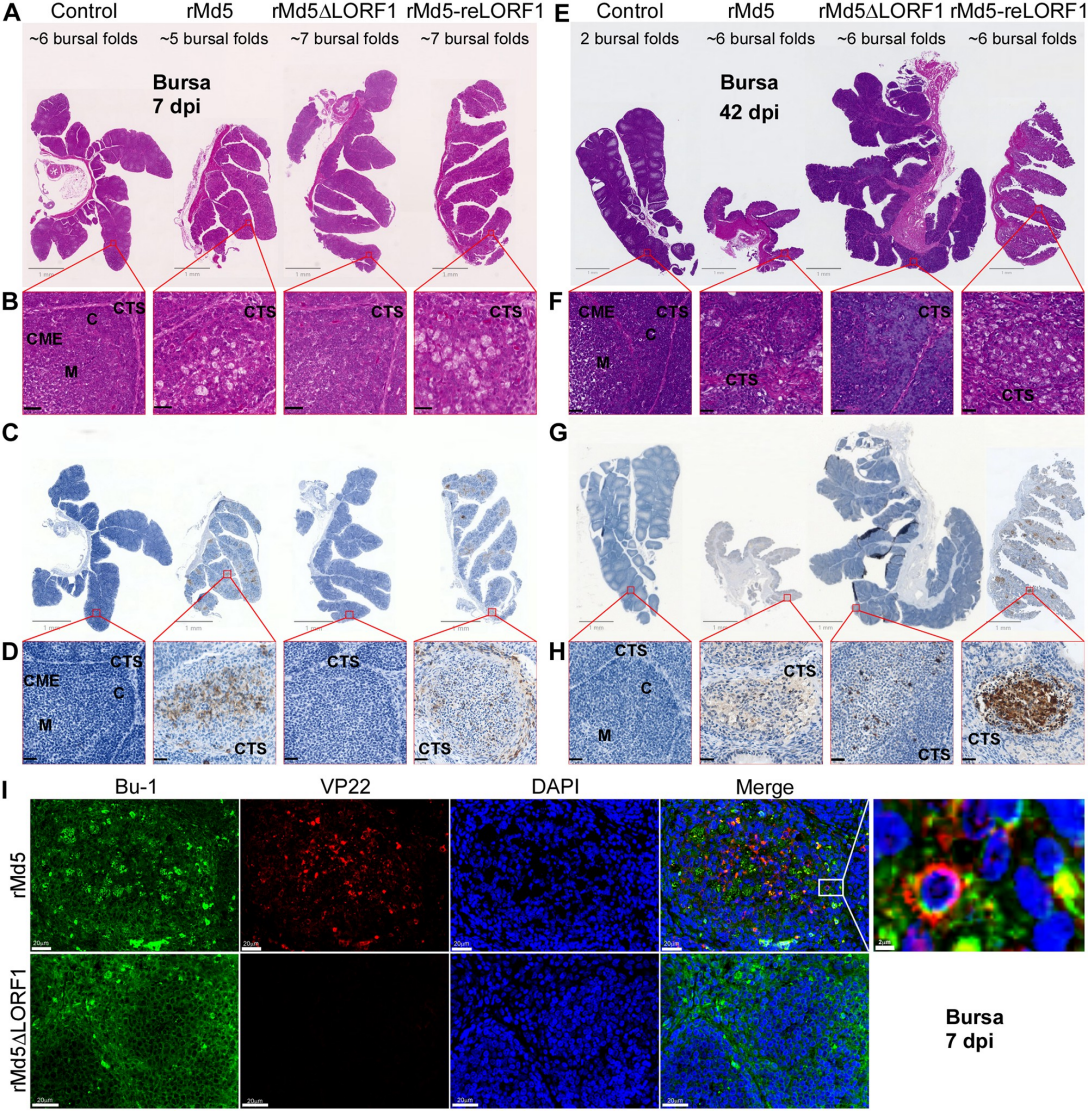
Histopathological examination of bursal tissues infected by recombinant viruses. The bursa tissues from chickens that were mock-treated or inoculated with 1,000 PFU of rMd5, rMd5ΔLORF1, or rMd5-reLORF1, and collected at 7 dpi (A-D) and 42 dpi (E-H). (A and E) Low-magnification images of HE-stained bursa tissues. (B and F) A higher magnification of the areas indicated by red boxes in A or E. (C and G) Low-magnification images of immunohistochemistry of bursa tissues. Bursal sections stained with anti-VP22 antibody and HRP-conjugated goat anti-mouse IgG antibody were visualized using DAB (brown), while the nuclei were stained with hematoxylin (blue). (D and H) Higher magnifications of the areas indicated by the red boxes in panels C or G. (I) Bursal sections at 7 dpi were stained with anti-Bu-1 and Alexa 488-conjugated goat anti-mouse IgG antibodies (green), followed by staining with anti-VP22 and Alexa 555-conjugated goat anti-mouse IgG antibodies (red). The nuclei were stained with DAPI (blue). CTS, Connective Tissue Septum; CME, Cortico-Medullary Epithelium; C, Cortex; M, Medulla. Scale bar: 1 mm (A, C, E, and G), 20 μm (B, D, F, and H).

To further analyze the effect of pLORF1 on viral replication in the bursa, H&E staining was performed on bursal tissues collected at 7 and 42 dpi. As shown in Fig 5A–5H, bursal tissues from the control group showed a well-preserved architectural structure characterized by folds and follicles, along with minimum inter-follicular connective tissue septa (indicated by “CTS”). The follicles comprised the medulla and cortex, separated by a single-celled layer of endodermal epithelium (marked as “M,” “C,” and “CME”). The medulla of the follicle contained loosely dispersed small lymphocytes, while the cortex was populated with large lymphocytes tightly packed with cells from the plasmacyte lineage. In contrast, bursal tissues from the rMd5 or rMd5-reLORF1 group displayed infiltration of reticular cells at 7 dpi (Fig 5A and 5B), and showed severely atrophied follicles with a noticeable increase in the thickness of the endodermal epithelium or fuzzy demarcated cortico-medullary regions, accompanied by lymphocytic depletion and necrosis in both the medulla and cortex at 42 dpi (Fig 5E and 5F). Reticular cells and heterophile infiltration, as well as severe inter-follicular fibroplasia, were also observed. Conversely, bursal tissues from rMd5ΔLORF1-infected chickens exhibited a normal follicular structure at 7 dpi (Fig 5A and 5B), and showed moderate follicular atrophy with lymphocytic infiltration and thickened inter-follicular connective tissue at 42 dpi (Fig 5E and 5F).

To investigate the lytic replication of MDV within the bursa of Fabricius, an immunohistochemistry assay was performed on bursal tissues infected with recombinant viruses by staining with an anti-VP22 antibody. The results showed that rMd5ΔLORF1-infected bursal tissues were negative following VP22 staining at 7dpi (Fig 5C and 5D) and showed a small amount of positive staining at 42 dpi (Fig 5G and 5H). Given that the bursa of Fabricius is where B cells develop and mature, to further examine whether pLORF1 affects MDV replication in B cells, bursal sections were immunostained with anti-Bu-1 (a B cell surface marker) and anti-VP22 antibodies. Confocal imaging revealed that VP22 was primarily colocalized with Bu-1 in rMd5-infected samples, but VP22 staining was negative in rMd5ΔLORF1-infected samples (Fig 5I).

Taken together, we concluded that pLORF1 is involved in viral replication in bursal B cells and in Md5-induced bursal atrophy, but not in tumor formation.

### Characterization of the LORF1 gene product

**Fig 6:**
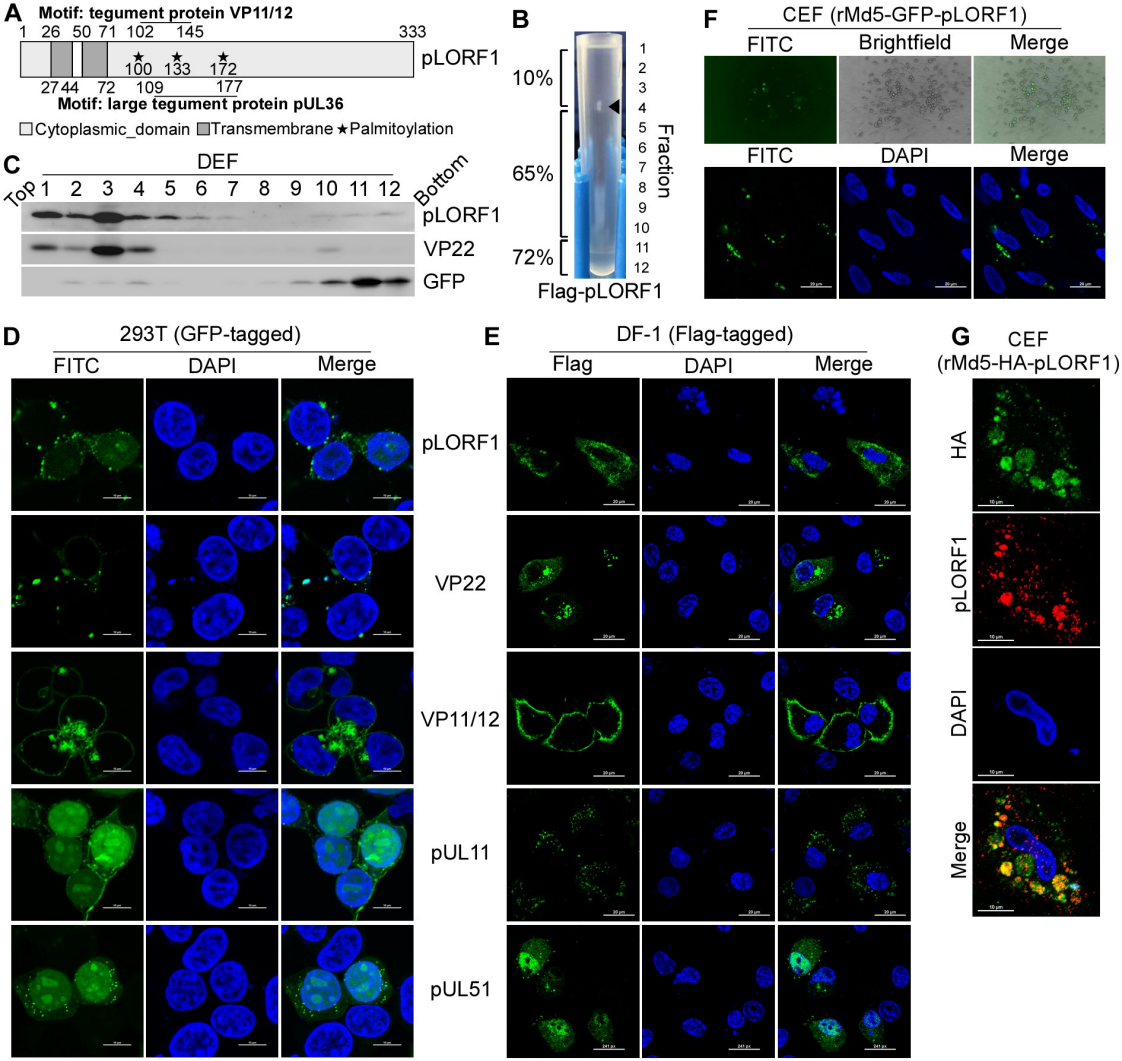
Analysis of the expression pattern of pLORF1. (A) Schematic diagram of pLORF1. (B and C) Membrane flotation analysis was conducted as described in the “Materials and Methods.” (B) Image of a 72%-65%-10% sucrose gradient of extracts from Flag-pLORF1-transfected DEF cells after ultracentrifugation. The mass of floated cellular membranes is marked by a black arrow. (C) Gradient fractions were analyzed using Western blotting with anti-Flag or anti-GFP antibodies. Fractions 1–5 correspond to the 65%-10% interface, which contains cellular membranes, while fractions 10–12 contain non-membrane-associated proteins. (D) Cellular localization of GFP-tagged pLORF1, VP22, VP11/12, pUL11, and pUL51. 293T cells were transfected with the indicated plasmids for 24 hours, then fixed and mounted in DAPI (blue). (E) Cellular localization of Flag-tagged pLORF1, VP22, VP11/12, pUL11, and pUL51. DF-1 cells were transfected with the indicated plasmids for 24 hours, followed by fixation and staining with anti-Flag antibody and Alexa 488-conjugated goat anti-mouse IgG antibody. (F) Cellular localization of pLORF1 in cells infected with rMDV. CEFs were infected with rMd5-GFP-pLORF1 for 3 days, fixed, mounted in DAPI, and analyzed by confocal microscopy. (G) Cellular localization of HA-tagged pLORF1 in cells infected with rMDV. CEFs were infected with rMd5-HA-pLORF1 for 3 days, fixed, and stained with anti-HA antibody and Alexa 488-conjugated goat anti-rabbit IgG antibody (green), followed by staining with anti-pLORF1 antibody and Alexa 555-conjugated goat anti-mouse IgG antibody (red). Scale bar: 10 μm (D and G); 20 μm (E and F).

To characterize the protein features of pLORF1, the functional domains were analyzed using InterPro (https://www.ebi.ac.uk/interpro/), motifs were identified with MotiFinder (https://www.genome.jp/tools/motif/), and lipid modifications were examined using GPS-Palm [53] (http://gpspalm.biocuckoo.cn/index.php). pLORF1 is predicted to contain two transmembrane domains, two motifs as observed in HSV-1 tegument proteins (VP11/12 and pUL36), and three palmitoylation sites, presented as a structural diagram (Fig 6A), indicating that pLORF1 may share similar features to a tegument protein. Studies show that tegument proteins of HSV-1, including VP22, VP11/12, pUL11, and pUL51, associate with cellular membranes [54–60]. The palmitoylation of pUL11 is essential for its targeting to the Golgi apparatus and membrane binding [60]. To examine the interaction of pLORF1 with membranes, lysates from DEF cells transfected with the Flag-tagged pLORF1 plasmid were analyzed using a membrane flotation assay. The lysates were mixed with sucrose to produce a 72% sucrose mixture, which was then loaded sequentially with 65% and 10% sucrose layers. Due to the buoyant density of membranes, ultracentrifugation resulted in the flotation of membrane-associated proteins to the interface between the 65% and 10% sucrose layers (Fig 6B). Western blot analysis of the membrane flotation gradients revealed that pLORF1 was present in fractions 1 to 5, along with a significant amount of VP22 and a small quantity of GFP protein detected (Fig 6C). The results suggest that pLORF1 expressed within transfected cells is associated with the cellular membranes.

Previous studies have shown that the tegument proteins VP22, VP11/12, pUL11, and pUL51 display a distinct punctate pattern in the cytoplasm of cells infected with HSV-1 or transfected with these proteins. This pattern resembles the trans-Golgi network (TGN) or TGN-derived vesicles, which are involved in tegument formation and envelopment [55–60]. To determine the subcellular localization of pLORF1, 293T cells were transfected with a plasmid expressing GFP-pLORF1. Confocal imaging revealed that GFP-pLORF1 displayed punctate perinuclear localization within the cytoplasm, similar to GFP-tagged VP22, VP11/12, pUL11, or pUL51 (Fig 6D). Similarly, it also showed punctate localization in the cytoplasm of DF-1 cells transfected with Flag-pLORF1 (Fig 6E). To further examine the cellular localization of pLORF1, recombinant viruses expressing GFP-tagged pLORF1 (rMd5-GFP-pLORF1) or HA-tagged pLORF1 (rMd5-HA-pLORF1) were generated (S5A Fig). The growth curves showed that both viruses proliferated at a rate comparable to that of the parental virus (S5B Fig). Moreover, CEFs infected with rMd5-GFP-pLORF1 displayed green fluorescent plaques, and confocal imaging revealed punctate localization of pLORF1 in the cytoplasm (Fig 6F). Furthermore, a similar localization pattern of HA-pLORF1 was also observed in rMd5-HA-pLORF1-infected CEFs by immunofluorescence analysis, co-immunostained with anti-HA and anti-pLORF1 antibodies (Fig 6G).

**Fig 7:**
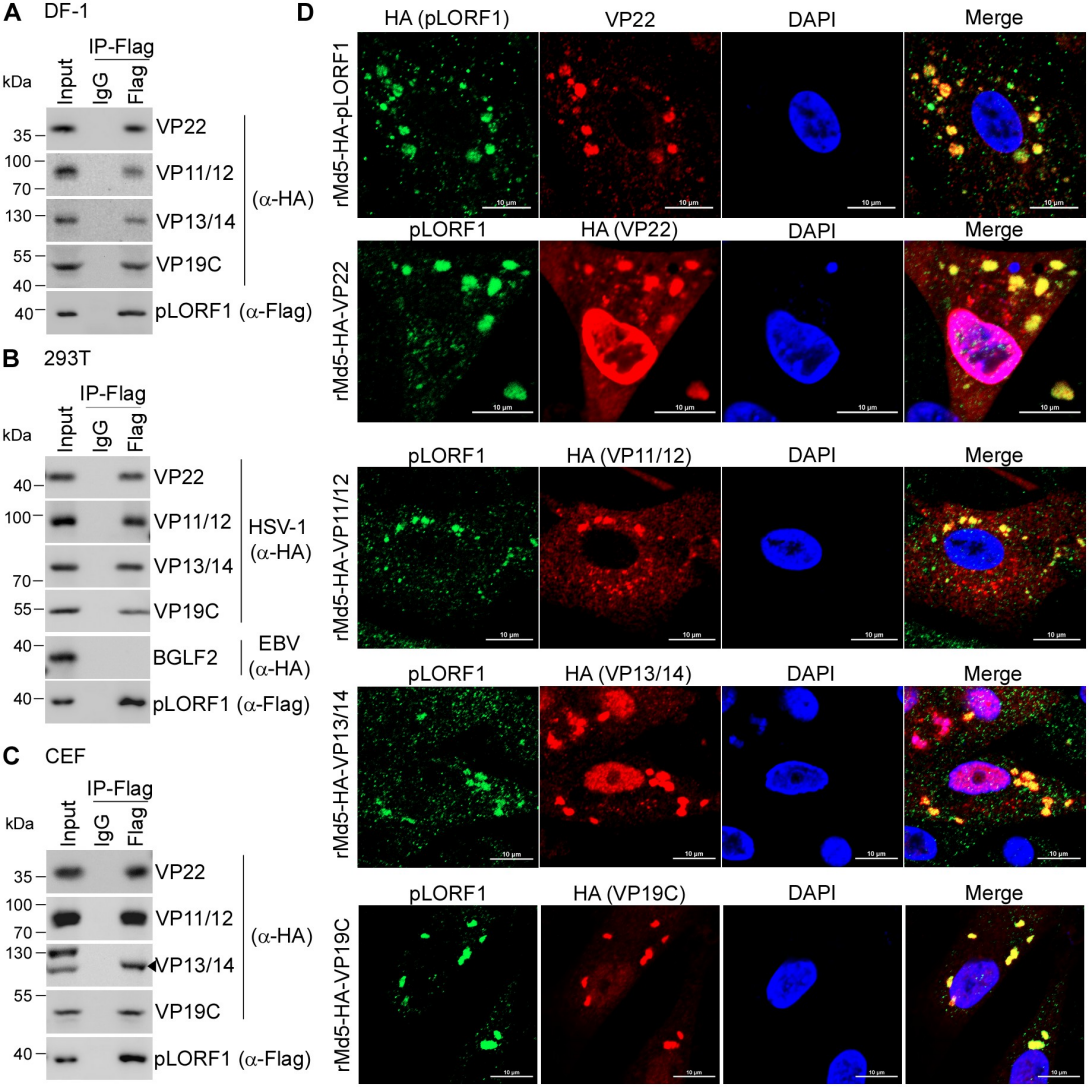
Analysis of interactions of pLORF1 with MDV structural proteins. (A-C) DF-1 cells (A) were co-transfected with Flag-tagged pLORF1 and HA-tagged MDV-1 proteins (VP22, VP11/12, VP13/14, or VP19C) for 36 hours. 293T cells (B) were co-transfected with Flag-tagged pLORF1 and HA-tagged HSV-1 proteins (VP22, VP11/12, VP13/14, or VP19C) or EBV BGLF2 for 36 hours. CEFs (C) were transfected with Flag-tagged pLORF1 for 6 hours and subsequently infected with rMd5-HA-VP22, rMd5-HA-VP11/12, rMd5-HA-VP13/14, or rMd5-HA-VP19C for 2 days. Co-immunoprecipitation assays were performed using whole cell lysates with anti-Flag antibody or control IgG. Immunocomplexes were analyzed by immunoblotting with anti-Flag and anti-HA antibodies. (D) Colocalization of pLORF1 with rMDV structural proteins. CEFs were infected with rMd5-HA-pLORF1, rMd5-HA-VP22, rMd5-HA-VP11/12, rMd5-HA-VP13/14, or rMd5-HA-VP19C for 3 days, then fixed and stained with anti-HA antibody and either Alexa 488 or Alexa 555-conjugated goat anti-rabbit IgG, followed by staining with anti-pLORF1 antibody and either Alexa 555 or Alexa 488-conjugated goat anti-mouse IgG.

It is well known that the tegument of herpesvirus is an organized structure formed by a dense network of interactions among tegument proteins, capsid proteins, and membrane proteins [61,62]. To investigate the interaction between pLORF1 and MDV structural proteins, co-immunoprecipitation assays were performed by co-transfecting Flag-tagged pLORF1 with HA-tagged VP22, VP11/12, VP13/14, or VP19C in DF-1 cells. The results showed the presence of tegument proteins (VP11/12, VP22, VP13/14) and capsid protein (VP19C) in the anti-Flag antibody-immunoprecipitated pLORF1 protein complex (Fig 7A). In addition, we confirmed the ability of pLORF1 to bind with homologous proteins by constructing plasmids expressing HA-tagged counterparts in the alphaherpesvirus HSV-1 and using the gammaherpesvirus EBV tegument protein BGLF2 as a negative control. The immunoprecipitation results showed that pLORF1 interacted with HSV-1 VP22, VP11/12, VP13/14, and VP19C, but not with EBV BGLF2 (Fig 7B). Furthermore, recombinant MDV viruses carrying HA-tagged VP22, VP11/12, VP13/14, and VP19C were successfully generated, respectively (S5C Fig). All recombinant viruses were rescued and exhibited growth rates comparable to that of the parental virus (S5D Fig). To further confirm the interaction between pLORF1 and these proteins during viral infection, CEFs were transfected with Flag-pLORF1, followed by infection with rMd5 expressing HA-tagged proteins, and then immunoprecipitated using an anti-Flag antibody. Results showed that HA-tagged VP22, VP11/12, VP13/14, and VP19C were detected in the anti-Flag (pLORF1) immunocomplex (Fig 7C). Finally, we examined the subcellular localization of pLORF1 and MDV proteins during infection. CEFs infected with rMd5-HA-pLORF1 or rMd5-HA-VP22 viruses were immunostained with anti-HA (pLORF1, green) and anti-VP22 (red) antibodies, or anti-pLORF1 (green) and anti-HA (VP22, red) antibodies. Confocal imaging showed that the endogenously expressed pLORF1 colocalized with VP22 and exhibited a punctate localization in the cytoplasm (Fig 7D and S6 Fig, upper two panels). Similar results were observed in CEFs infected with rMd5-HA-VP11/12, rMd5-HA-VP13/14, and rMd5-HA-VP19C, where pLORF1 exhibited punctate colocalization with VP11/12, VP13/14, and VP19C in the cytoplasm (Fig 7D and S6 Fig, bottom three panels). Taken together, these results suggest that pLORF1 shares similar features to certain tegument proteins.

## Discussion

Marek’s disease (MD), caused by Marek’s disease virus (MDV), poses significant economic challenges to the poultry industry worldwide due to its highly contagious and oncogenic nature [5]. Despite the availability of vaccines, MDV continues to evolve, necessitating the development of improved next-generation vaccines. However, our understanding of the mechanisms underlying MDV-induced tumorigenesis and immunosuppression remains incomplete. With MDV encoding more than 100 genes, most of which have unknown functions, exploring the roles of these genes is crucial. Among these genes, MDV LORF1 is unique to MDV-1, lacking homologs in other herpesviruses [30]. However, its function in MDV pathogenesis remains largely unknown [47]. In this study, we investigated the function of LORF1 in MDV infection both *in vitro* and *in vivo* using genetically modified Md5-deficient in pLORF1 expression. Our findings suggest that pLORF1 is involved in viral replication and MDV-induced bursal atrophy, but not in tumor development.

The complete genome of the MDV-1 Md5 strain was cloned as an infectious BAC using a visible selection marker (RFP) and the BAC vector pBeloBAC11 (Fig 1). Previously reported MDV-BAC constructions exclusively relied on the expression of the *Eco-gpt* gene and selection media containing mycophenolic acid, xanthine, and hypoxanthine to enrich recombinant viruses [9–18]. In this study, we introduced the RFP expression cassette for the first time in MDV-BAC construction, which simplifies the purification of positive clones. Additionally, the transfer vector also includes two 34-bp loxP sites flanking the BAC and RFP fragment, enabling the rapid excision of BAC vector sequences using the Cre/loxP homologous recombination system (Fig 1 and S1 Fig). Transfection of the DNA prepared from BAC clones and the Cre expression vector in CEFs resulted in the rescue of infectious viruses with plaque morphology and growth kinetics indistinguishable from those of the parental Md5 virus (Fig 2B and 2C). In animal studies, BAC-derived rMd5 viruses exhibited high pathogenicity and oncogenicity, as well as horizontal transmissibility, comparable to the parental Md5 virus *in vivo* (Fig 2 and S1 Fig). During the experiments, we observed that the majority of chickens infected with rMd5 developed gross tumors and/or bursal atrophy, with a few exceptions (S1D Fig). Indeed, a previous report showed that 14.3% of maternal antibody-positive chickens and 2.6% of negative chickens showed no bursal atrophy when infected with the vv+MDV 648A strain [52]. In addition, H&E staining revealed lymphocyte infiltration of rMd5-infected liver tissues from chickens without obvious tumor nodules collected at 28 dpi (S1F Fig), consistent with previous reports that 20% of Md5-infected chickens developed obvious tumors [27,63].

Using the Red-mediated recombination system, we constructed the pLORF1-deficient virus, rMd5ΔLORF1, by creating premature stop codons right after the translation start site due to a 2-bp frameshift deletion within the LORF1 gene, along with the revertant virus, rMd5-reLORF1 (Fig 3A). MD pathogenesis involves three main stages: an early cytolytic phase (2–7 dpi) mainly targeting and infecting B lymphocytes [64,65], activating CD4+ T cells; a latent phase with no viral antigen expression; and a second cytolytic phase leading to rapid proliferation of latently infected and transformed lymphocytes, resulting in tumor formation in tissues [5,66,67]. In animal studies, we observed that the loss of pLORF1 expression attenuated rMd5-induced pathogenesis, delaying weight loss and bursal atrophy compared to the parental and revertant viruses (Fig 4A–4C and S3 Fig). Interestingly, the viral load of rMd5ΔLORF1 was notably lower than that of rMd5 or rMd5-reLORF1 in the bursa, but not in the spleen by 35 dpi (Fig 4D). The loss of pLORF1 did not affect rMd5 replication *in vitro* (Fig 3E). There was almost no replication of rMd5ΔLORF1 in the bursa and no damage to bursal structure at the early stage of infection (7 dpi) (Fig 5A–5D), but only moderate replication of rMd5ΔLORF1 concomitantly with lymphocytic infiltration at the late stage of infection (42 dpi) (Fig 5E–5G). The bursa of Fabricius is a primary lymphoid organ in birds responsible for amplifying and differentiating B lymphoid progenitors within its follicular microenvironment. Our study showed that the loss of pLORF1 abolished the capability of rMd5 to replicate in bursal B cells at 7 dpi (Fig 5I).

Efficient virus replication in the host is a prerequisite for disease and tumor formation. However, the low level of viral replication in the bursa due to pLORF1 deficiency did not affect the ability of rMd5 to induce lymphomas (Fig 5E and 5F, and S4 Fig). As previously reported, in the absence of B cells, MDV can readily replicate in macrophages [68,69], T cells [65,70,71], and natural killer cells [72], compensating for the loss of B cells during lytic replication. MD pathogenesis and tumor formation were not altered in infected chickens that lack mature and peripheral B cells [73]. These results suggest that pLORF1 is essential for early cytolytic infection in bursal B cells, but not for tumor formation. Integration of the MDV genome into latently infected and transformed cells has been shown to be essential for effective tumor formation [74,75]. Therefore, indistinguishable viral loads (genome copies) of recombinant viruses in the bursa at 42 dpi may be caused by infiltration with lymphocytes bearing viral genomes.

To date, there is only limited information available regarding the MDV-1 unique gene LORF1. Based on prediction data, pLORF1 contains two transmembrane domains, two tegument motifs (VP11/12 and pUL36), and at least three palmitoylation sites (Fig 6A). Importantly, we found that pLORF1 can bind to the cellular membrane, has a punctate distribution in the cytoplasm, and interacts with tegument proteins (VP22, VP11/12, VP13/14) and the capsid protein (VP19C) (Figs 6 and 7). The ability of tegument proteins to target membranes helps transport viral components and recruit essential host cell factors to the specific assembly site for forming new infectious particles [76–78]. Previous studies have shown that the tegument proteins VP22, VP11/12, pUL11, and pUL51 display a distinct punctate pattern in the cytoplasm of cells. This punctate localization resembles the trans-Golgi network (TGN) and TGN-derived vesicles [55–57,78], where tegument proteins direct HSV-1 virion assembly by acquiring the envelope and nucleocapsid [79–81], which have been extensively studied with HSV-1 proteins VP22, VP11/12, pUL11, and pUL51. Given that palmitoylation enhances the hydrophobicity of proteins, resulting in alterations to protein conformation, stability, membrane association, localization, and binding properties [82], it is possible that pLORF1 binds to TGN membranes through its transmembrane domains and palmitoylation, thereby recruiting nucleocapsids and tegument proteins for viral packaging. Here, we would also like to note that the conserved amino acid residues shared between pLORF1 and the tegument proteins VP11/12 or pUL36, which have not yet been characterized in terms of any biological function, warrant further investigation to elucidate the specific roles of these motifs. In addition, the role of pLORF1 during the specific stage of virus replication needs to be further explored as well.

In this study, we found that the MDV-1 unique gene LORF1 is a nonessential gene for replication, but it plays an important role in bursal atrophy, indicating its potential for the development of a gene deletion vaccine for MD. The Meq deletion virus (rMd5ΔMeq) conferred superior protection against challenge with vv+ MDV strains, making it a promising vaccine candidate [28,29]. However, rMd5ΔMeq retains the ability to induce lymphoid organ atrophy and lower body weights (Fig 4C and S4B Fig) in maternal antibody-negative chickens [83], rendering it unsuitable for approval as a vaccine. Attempts to eliminate rMd5ΔMeq-induced lymphoid organ atrophy through serial cell culture passages led to an attenuated virus with decreased protective efficacy [84]. An alternative strategy involved introducing known secondary mutations in the MDV genome by deleting or mutating a second gene related to virus replication. Studies have demonstrated that introducing a point mutation in UL5 (a helicase-primase subunit), codon deoptimization of UL54 (a multifunctional ICP27-like protein), ablation of UL23 (thymidine Kinase), deletion of pp38 (a phosphorylated protein), LORF9 (a unique MDV gene), or vIL-8 (an interleukin-8 homolog) led to reduced lymphoid organ atrophy and provided partial or comparable protection to commercial vaccines against vv or vv+ MDV strains [63,85–89]. These double deletion viruses exhibited comparable replication capacity to the parental virus *in vitro* but were significantly less replicative and pathogenic *in vivo*, while inducing varying degrees of immune response. This suggests that the protective efficacy of MD vaccines is deeply associated with their replication *in vivo*. Our findings suggest that pLORF1 deficiency inhibits MDV replication in the bursa, preventing early-stage bursal atrophy and body weight loss. Defective pLORF1 expression may prevent immunosuppressive effects while inducing maximum disease protection by the rMd5ΔMeq virus. Further studies will validate the efficacy of the Meq&LORF1-deleted vaccine.

## Materials and methods

### Ethics statement

All animals and experimental protocols were approved by the Animal Welfare and Ethics Committee of Nanjing Agricultural University (SYXK(Su)2021-0086).

### Cells, viruses, and reagents

293T and DF-1 cells were cultured in Dulbecco’s Modified Eagle’s Medium (DMEM; Gibco) supplemented with 8% fetal bovine serum (FBS; PAN Biotech, Germany) and 1% penicillin and streptomycin at 37°C with 5% CO_2_. Chicken embryo fibroblasts (CEFs) were prepared from 10-day-old specific-pathogen-free (SPF) chicken embryos and maintained in DMEM containing 5% FBS (Gibco, USA). Duck embryo fibroblasts (DEFs) were prepared from 10-day-old duck embryos and cultured in DMEM containing 10% FBS (Gibco, USA). The virulent MDV Md5 strain (GenBank: AF243438.1; kindly provided by Dr. Xunhai Zhang, Anhui Science and Technology University, China) was propagated in CEFs.

### Antibodies

A mouse anti-pLORF1 polyclonal antibody was generated by using the pET30a vector to express the C-terminal portion of pLORF1 (aa 174 to 333) as a fusion protein with (His)_6_ tags at both ends, which was purified using Ni-NTA agarose (Sangon Biotech, China) and injected into BALB/c mice to generate antisera. A rabbit anti-HA antibody was purchased from Cell Signaling Technology (USA). Mouse anti-Flag, horseradish peroxidase (HRP)-conjugated goat anti-rabbit and anti-mouse IgG antibodies were purchased from Sigma-Aldrich (USA). Mouse anti-Bu-1, Alexa Fluor-conjugated goat anti-mouse and anti-rabbit IgG antibodies were purchased from Thermo Fisher Scientific (USA). A mouse anti-VP22 monoclonal antibody (clone 3F7) was provided by Dr. Hongjun Chen from the Shanghai Veterinary Research Institute in China.

### Construction of transfer vectors

A 2.1-kbp *Not*I-*Bam*HI fragment and a 3.0-kbp *Hin*dIII-*Kpn*I fragment flanking the MDV-1 US2 gene were amplified by PCR using the primers listed in Table 1. Both fragments were subsequently cloned into pBluescript II KS(+) to yield pBlue-US2A-US2B. A 2.5-kbp *Sal*I fragment of pBS302 (Addgene, USA) was amplified by PCR using primers provided in Table 1 and then circularized as pBS302-CB. To construct pBS302-BAC-RFP, a 6.4-kbp *Sal*I fragment of pBeloBAC11 (NEB, USA) and a 2.1-kbp *Not*I-*Bam*HI fragment of the NEDD8 HDR plasmid (Santa Cruz, USA) containing the red fluorescent protein (RFP) gene and the puromycin resistance gene (both sites were blunt-ended) were cloned into the *Sal*I and *Hpa*I sites of pBS302-CB, respectively. To generate pBlue-US2-BAC-RFP, a *Not*I fragment of pBS302-BAC-RFP containing the BAC vector and RFP & puromycin cassette (the *Not*I sites were blunt-ended) was cloned into the *Sma*I site in pBlue-US2A-US2B.

**Table 1:** Primers used in this study.

Name	Sequence (5’→3’)
US2A-F	GTGCGGCCGCGTGTTTGAATACTGGAGAC
US2A-R	CTGGATCCCGACGGTAGTCATTAGCTG
US2B-F	CGAAGCTTTTTGGCAAAACGGAATAGGTCTGCAGC
US2B-R	GCGGTACCGCGGCCGCAATATGAATCTCTAAAACTTC
pBS302-CB-F	AAAGTCGACGAGCTCGATATCTGGTGTCCCCGAGGATCCGGA
pBS302-CB-R	CTGGTCGACGCATGCCCCGGGGGCTGCAGGTCGAGGGACCTA
US2Aj-F	TTATCAACTGCCACATTCACATCCG
US2Aj-R	TCACCCTTGTTACACCGTTTTCCAT
US2Bj-F	TACGGCAGCAAAGCCTTCATCAACC
US2Bj-R	AATCCGCAGAACGCAACAAGAGCAG
RFP-F	GGGCATCCCCGACTTCTTTA
RFP-R	GGCATCTTGAGGTTCTTAGCG
SopA-F	TAGGTGAAGCAGCGGATTTAGT
SopA-R	CGTCCTTTTCCCCAAGATAGA
VP22-F	AATAGTTTGCGGGCACAGG
VP22-R	AATTCGCTTTCAGTACGGCTC
ICP4-F	CCTCTTCATCTTCCTCCTCT
ICP4-R	TGTCACCTGAATATCATTGC
actin-F	GAGACCTTCAACACCCCAGCCATG
actin-R	GCGACGTAGCACAGCTTCTCCTTG
ΔLORF1kana-F	ACATGTTTGATTTATCATTGAGTAATTATAGTTGCGTCTACACCATTGCATTAGGTAGTCTAGGGATAACAGGGTAATCGATTTATTCAACAAAGCCA
ΔLORF1kana-R	TTAATGTCGTGGCAAACCACGACTACCTAATGCAATGGTGTAGACGCAACTATAATTACTGCCAGTGTTACAACCAATTAA
reLORF1Kana-F	ATGTTTGATTTATCATTGAGTAATTATAGTTGCGTCTATTCACCATTGCATTAGGTAGTCTAGGGATAACAGGGTAATCGATTTA
reLORF1Kana-R	TTAATGTCGTGGCAAACCACGACTACCTAATGCAATGGTGAATAGACGCAACTATAATTAGCCAGTGTTACAACCAATTA
LORF1 seq-F	CGCCTAGCGTAGCGTTCC
LORF1 seq-R	GATCCGACCTTAATATCTG
ΔMeq (MDV076) kana-F	TATACCAGGGAGAAGGCGGGCACGGTACAGGTGTAAAGAGTGATCCGCATTGTGACTCTCTAGGGATAACAGGGTAATCGATTT
ΔMeq (MDV076) kana-R	GGCATAGACGATGTGCTGCTGAGAGTCACAATGCGGATCACTCTTTACACCTGTACCGTGGCCAGTGTTACAACCAATTAACC
ΔMeq (MDV005) kana-F	GGCATAGACGATGTGCTGCTGAGAGTCACAATGCGGATCACTCTTTACACCTGTACCGTGTAGGGATAACAGGGTAATCGATTT
ΔMeq (MDV005) kana-R	TATACCAGGGAGAAGGCGGGCACGGTACAGGTGTAAAGAGTGATCCGCATTGTGACTCTCGCCAGTGTTACAACCAATTAACC
Meq-F	CAATACTTTCGGGTCTGTGG
Meq-R	CGAGTCTAAGCTACACGGTAAG
GFP-LORF1Kana-F	CGACATGTTTGATTTATCATTGAGTAATTATAGTTGCGTCTATTCACCATTCCGGACTTGTACAGCTCGTC
GFP-LORF1Kana-R	CTAAACAATGTATAGTTAATGTCGTGGCAAACCACGACTACCTAATGCAGCCACCATGGTGAGCAAGG
HA-LORF1Kana-F	CGACATGTTTGATTTATCATTGAGTAATTATAGTTGCGTCTATTCACCATGAATTCAGCGTAATCTGGAACATCGTATGGGTACATGGTGGCTAGGGATAACAGGGTAATCGATTTATTC
HA-LORF1Kana-R	CTAAACAATGTATAGTTAATGTCGTGGCAAACCACGACTACCTAATGCAGCCACCATGGATTACAAGGATGACGACGATAAGGAATTCGAGCTCGCCAGTGTTACAACCAATTAACC
HA-VP22Kana-F	CCAAGGGAACGACGCCGTTCCGATTTCCGCCTTTCAGAATCCCCCATGAATTCAGCGTAATCTGGAACATCGTATGGGTACATGGTGGCTAGGGATAACAGGGTAATCGATTTATTC
HA-VP22Kana-R	GAAGGTGCACTTGTTCATATCTTACTGTTTAATATTATATCTTAGTTATCGCCACCATGTACCCATACGATGTTCCAGATTACGCTGAATTCGCCAGTGTTACAACCAATTAACC
HA-VP11/12Kana-F	CTCGGTCAAACAGTTCCAACGACTGTTCAGAAGAGCTGAGCCGCTTCATGAATTCAGCGTAATCTGGAACATCGTATGGGTACATGGTGGCTAGGGATAACAGGGTAATCGATTTATTC
HA-VP11/12Kana-R	GTACTATATTTCAGTTTTCTCTGTAGTGTGTCCATCCATATGGGTTATAGCCACCATGTACCCATACGATGTTCCAGATTACGCTGAATTCGCCAGTGTTACAACCAATTAACC
HA-VP13/14Kana-F	CGTTGATTTTGACCAGGATGTCCATACCGATGCATAGAAGGCATTTGCATGAATTCAGCGTAATCTGGAACATCGTATGGGTACATGGTGGCTAGGGATAACAGGGTAATCGATTTATTC
HA-VP13/14Kana-R	CGGCGTAGTTTTGATAACAGTATGCTGGTAGCACATTCCACCGAAGAGCCACCATGTACCCATACGATGTTCCAGATTACGCTGAATTCGCCAGTGTTACAACCAATTAACC
HA-VP19CKana-F	CAGTATTTTTACTAGTAGCTAATTTCGATTTCCGTCAATTATCTACATCGCCACCATGTACCCATACGATGTTCCAGATTACGCTGAATTCTAGGGATAACAGGGTAATCGATTTATTC
HA-VP19CKana-R	GGATACAGACTGTAGTATTGTGTTTCGTGCGATCGTAAGAGTGGTTTCATGAATTCAGCGTAATCTGGAACATCGTATGGGTACATGGTGGCGCCAGTGTTACAACCAATTAACC

### Construction of Md5 BAC

CEFs were transfected with linearized pBlue-US2-BAC-RFP using jetPRIME transfection reagent (Polyplus, France). At 8 hours post-transfection, the CEFs were inoculated with MDV Md5 at 0.01 PFU/cell. At 3 days post-inoculation, monolayers were harvested and serially diluted on fresh CEFs in 96-well plates. At 7 days post-inoculation, a single red fluorescent plaque was picked from the well. After six rounds of plaque purification, the recombinant virus Md5-BAC-RFP DNA was isolated from infected CEFs using the Hirt method as previously described [90], and then electroporated into *E*. *coli* DH10B cells. Chloramphenicol-resistant colonies were analyzed for the integrity of Md5 genomes by restriction fragment length polymorphism (RFLP) analysis. The BAC DNA was digested with *Eco*RI, *Bam*HI, *Hin*dIII, or *Sac*I and separated on 0.8% agarose gels. The profiles obtained were compared to the simulated profiles generated by SnapGene software. For the infectivity of pMd5, BAC DNA was transfected into CEFs, and the growth of the rescued virus rMd5-BAC-RFP was observed with the aid of a fluorescence microscope.

### Removal of BAC sequence

The loxP-flanked BAC and RFP & puromycin sequences within the infectious clones were removed by co-transfection of pMd5 DNA and a Cre recombinase expression vector (pcDNA3-Cre: GFP). Removal of the exogenous sequences was confirmed by analyzing recombinant virus rMd5 stocks using PCR amplification. Genomic regions of viral, BAC, RFP, and junctional DNA were amplified using the primers provided in Table 1.

### Construction of recombinant viruses

To generate the recombinant virus rMd5ΔLORF1, in which LORF1 harbored a frameshift due to a 2-bp deletion, the two-step Red-mediated mutagenesis procedure was conducted using *E*. *coli* GS1783 (kindly provided by Dr. Nikolaus Osterrieder, Freie Universität Berlin, Germany) containing pMd5, following the previously described protocol [51], with the primers listed in Table 1. The revertant virus rMd5-reLORF1, the full-length Meq-deleted virus rMd5ΔMeq, and several tagged viruses (rMd5-GFP-pLORF1, rMd5-HA-pLORF1, rMd5-HA-VP11/12, rMd5-HA-VP22, rMd5-HA-VP13/14, and rMd5-HA-VP19C) were generated using the same strategy, except for the primers listed in Table 1.

### Growth curve analysis

The growth curves of recombinant viruses were determined as previously described [9]. Briefly, CEFs pre-seeded in 6-well plates were infected with 100 PFU of each virus. At 0, 1, 2, 3, 4, and 5 dpi, the infected cells were trypsinized and serially diluted on fresh CEFs pre-seeded in 6-well plates, and the plaques were counted at 7 dpi.

### Chicken experiment

One-day-old SPF chickens were randomly divided into groups (n = 25) and intraperitoneally injected with 1,000 PFU of either Md5 or rMd5/14-20, and contact chickens (n = 3) were kept in the same isolator as the Md5 or rMd5 group. A negative control group (n = 10) was injected intraperitoneally with DMEM. The birds were monitored daily, and mortality rates were recorded. To assess viral pathogenesis and histopathology (gross and histological lesions), one bird from the control group and two birds from the Md5 or rMd5 group were randomly selected and euthanized at 28 dpi or 42 dpi. The spleens and bursas were dissected and photographed. The liver and heart tissues were dissected, fixed in neutral buffered 4% paraformaldehyde, processed routinely for paraffin embedding, sectioned, and stained with hematoxylin-eosin (H&E). The experiment was terminated at 54 dpi. To detect the transmission of rMd5, three contact birds from the Md5 or rMd5 group were euthanized, the spleen tissues were collected for DNA preparation, and the liver tissues were dissected for H&E staining. Meanwhile, moribund and surviving birds were humanely euthanized and assessed for gross and histological lesions.

To characterize recombinant rMd5ΔLORF1, a total of 149 one-day-old SPF chickens were randomly divided into five groups: rMd5ΔLORF1 group (35 chickens, 20 for survival rate, 15 for sampling), rMd5-reLORF1 group (35 chickens, 20 for survival rate, 15 for sampling), rMd5ΔMeq group (35 chickens, 20 for survival rate, 15 for sampling), rMd5 group (28 chickens, 20 for survival rate, 8 for sampling), and a negative control group (16 chickens, 8 for survival rate, 8 for sampling). The first four groups were intraperitoneally injected with 1,000 PFU of each virus, while the control group was injected with DMEM. Chickens were monitored daily, and mortality rates were recorded from 5 to 60 dpi. At 7, 14, 21, 28, 35, and 42 dpi, one bird from the control or rMd5 group and two birds from the rMd5ΔLORF1, rMd5-reLORF1, or rMd5ΔMeq groups were humanely euthanized. The spleens and bursas were dissected for DNA preparation, and the latter were also photographed and weighed. Liver and bursal tissues were fixed for histopathological analysis.

### Immunohistochemistry (IHC)

Bursas were harvested at 7 and 42 dpi, and fixed in 4% paraformaldehyde. Tissue sections underwent microwave antigen repair and blocking, followed by overnight incubation at 4°C with either anti-VP22 antibody or Bu-1 antibodies. The sections were then incubated for 50 minutes at room temperature with HRP-conjugated goat anti-mouse IgG antibody or Alexa-conjugated goat anti-mouse IgG antibodies. HRP was visualized using the DAB Kit (Wuhan Servicebio Technology, China), and nuclei were stained with either hematoxylin or DAPI. Images were captured using a microscope scanner (Grundium Ocus 40, Finland) or a confocal microscope (Nikon Eclipse Ti, A1, Japan).

### Plasmids

To generate GFP-tagged pLORF1, VP22 (encoded by the UL49 gene), VP11/12 (encoded by the UL46 gene), pUL11, or pUL51, HA-tagged VP22, VP11/12, VP13/14 (encoded by the UL47 gene), or VP19C (encoded by the UL38 gene), and 2xFlag-tagged pLORF1, DNA fragments were amplified from the Md5 genome and cloned into pEGFP-C1, pcDNA3-HA, and pcDNA3-2xFlag using their respective restriction sites. HSV-1 VP22, VP11/12, VP13/14, and VP19C were amplified from the F strain genome (GenBank: KM222724.1) and cloned into the pCAGGS-N-HA vector. EBV BGLF2 (encoded by the UL16 gene; GenBank: NC_007605.1) was synthesized and cloned into the pCAGGS-N-HA vector by GenScript (China). The primers used in this study are listed in S2 Table.

### Membrane flotation gradient centrifugation

The protocol for the analysis of membrane-associated proteins by sucrose density gradient flotation was performed as previously described [56,57]. Briefly, DEF cells were transfected with pcDNA3-2xFlag-pLORF1, pcDNA3-2xFlag-VP22, or pEGFP-C1 for 24 hours. The cells were collected and resuspended in a hypotonic lysis buffer before being lysed by passing them through a 25-gauge needle. Post-nuclear supernatants (PNS) were obtained through low-speed centrifugation. The PNS obtained was loaded onto the bottom of a non-continuous 72%-65%-10% sucrose gradient and centrifuged at 150,000 × g at 4°C for 18 hours. The gradients were fractionated from the bottom and then mixed with trichloroacetic acid (TCA) for precipitation. After centrifugation, the pellets were washed once with ethanol, solubilized with 2×SDS sample buffer, and analyzed by Western blot.

### Confocal imaging

293T or DF-1 cells were pre-seeded on coverslips and transfected with GFP-tagged or Flag-tagged plasmids for 24 hours. CEFs were infected with MDV for 3 days. The coverslip cells were fixed and permeabilized with 4% formaldehyde and 0.1% Triton X-100 at 37°C for 30 minutes, washed with glycine-PBS, and blocked with 3% BSA in PBS at 37°C for 30 minutes. They were incubated with a primary antibody (1:200) at 37°C for 1 hour, followed by a secondary antibody (1:500) for 30 minutes. Nuclei were visualized with DAPI in an anti-fade DABCO solution and examined using a confocal microscope.

### Co-immunoprecipitation and Western blotting

293T or DF-1 cells were co-transfected with pcDNA3-2xFlag-LORF1 and HA-tagged plasmids for 36 hours. CEFs were first transfected with pcDNA3-2xFlag-LORF1 and infected with HA-tagged rMDV for 48 hours. Cells were collected in lysis buffer (50 mM Tris-HCl [pH 7.5], 150 mM NaCl, 1 mM EDTA, and 0.5% NP-40 supplemented with protease inhibitor cocktails) and incubated with an anti-Flag antibody, along with protein A/G magnetic beads, for 4 hours at 4°C. Mouse IgG was used as a negative control. The beads were washed five times with ice-cold lysis buffer. The precipitation was mixed with 2×SDS sample buffer and boiled for 10 minutes at 98°C. After centrifugation, the supernatant was used for Western blot analysis.

### Statistical analysis

All experiments were performed at least three times unless otherwise noted. The data are presented as the means ± standard deviations (SD). Statistical significance between groups was determined by a Student’s *t*-test using GraphPad Prism 7.0 software. A *p*-value of < 0.05 was considered statistically significant.

## Supporting information

S1 Table Bursa/body weight ratios in experimental chickens at different time points post-inoculation. (DOCX)

S2 Table Primers used for the construction of expression plasmids. (DOCX)

S1 Fig Analysis of the infectious clone pMd5 BACs and BAC-derived viruses. (A) The image shows a simulated experiment using the SnapGene software. Md5 or pMd5 BAC genomes are digested with *Eco*RI, *Bam*HI, *Hin*dIII, or *Sac*I on a 0.8% agarose gel. The bands marked with asterisks indicate the insertion of the transfer vector. (B) Recombinant viruses rMd5-BAC-RFP clones 14–4 and 14–20 were generated by transfecting CEFs with pMd5 DNA. At 5 days post-transfection, plaques expressing RFP were observed under fluorescence microscopy. (C) Recombinant viruses rMd5/14-4 and rMd5/14-20 were obtained by co-transfecting CEFs with pMd5 and Cre expression plasmid. The junction fragments (US2Aj and US2Bj), inserted genes (RFP and sopA), and viral genes (VP22) in mock-, Md5-, rMd5-BAC-RFP, and rMd5-infected CEFs at 3 dpi were amplified by PCR. (D-G) The animal experiment was performed as described in “Materials and Methods.” (D) Images of the bursas and spleens from chickens of the Md5 or rMd5 group at 28 dpi. (E) Images of the livers from chickens in each group at 42 dpi. White arrows indicate tumor nodules in the livers. (F) Liver tissues infected with rMd5 at 28 dpi were stained with H&E. (G) Liver tissues from contact chickens at 54 dpi in each group were stained with H&E. Scale bar: 20 μm (F and G).
(TIF)

S2 Fig Construction of the Meq-deleted virus. (A) The linear MDV-1 Md5 genome and a portion of the repeat regions (TRL and IRL) encoding genes LORF11 to vIL8 are depicted. (B) Schematic diagrams of the rMd5ΔMeq virus with a full-length deletion of the Meq gene. (C) DNA analysis of pMd5 and pMd5ΔMeq genomes. DNAs purified from BACs containing *E*. *coli* were digested with *Hin*dIII and separated on a 0.8% agarose gel. The bands that appeared after the deletion of the Meq gene are marked with arrows. (D) PCR amplification result of the Meq locus in pMd5 or pMd5ΔMeq. (E) CEFs infected with 100 PFU of rMd5 or rMd5ΔMeq were harvested at the indicated time points and subsequently seeded onto fresh CEFs. Viral plaques were counted at 7 dpi. Each value represents the mean of two independent duplicates.
(TIF)

S3 Fig Loss of pLORF1 attenuates MDV-induced bursal atrophy. One-day-old SPF chickens were intraperitoneally inoculated with 1,000 PFU of rMd5, rMd5ΔLORF1, or rMd5-reLORF1, and DMEM as a negative control. The chickens were kept in isolators for daily monitoring of MD symptoms or death. (A) Images of bursas of four representative dead chickens from each group at the indicated time points. (B) The histogram of the body weights of chickens in each group at 7, 14, 21, 28, 35, and 42 dpi. Results represent the mean value, with error bars indicating the standard error of the mean. (C) Images of chicken bursas from each group at the indicated time points, as in B.
(TIF)

S4 Fig Histopathological examination of chicken liver tissues infected with recombinant viruses. The liver tissues from chickens were mock-treated or inoculated with 1,000 PFU of rMd5, rMd5ΔLORF1, or rMd5-reLORF1 and euthanized at 35 dpi. (A) Focal infiltration of lymphoid tumor cells in the liver tissues is highlighted by white arrows (H&E). (B) A higher magnification of areas indicated by red boxes in A. Scale bar: 1 mm (A), 20 μm (B).
(TIF)

S5 Fig Construction of recombinant viruses expressing GFP-tagged or HA-tagged viral proteins. (A and C) RFLP analysis of *Hin*dIII-digested BAC DNAs, including pMd5, pMd5-HA-pLORF1, pMd5-GFP-pLORF1, pMd5-HA-VP22, pMd5-HA-VP11/12, pMd5-HA-VP13/14, and pMd5-HA-VP19C. (B and D) The growth curve of recombinant viruses, including rMd5, rMd5-HA-pLORF1, rMd5-GFP-pLORF1, rMd5-HA-VP22, rMd5-HA-VP11/12, rMd5-HA-VP13/14, and rMd5-HA-VP19C. Each value represents the mean of two independent duplicates.
(TIF)

S6 Fig Colocalization of pLORF1 with MDV structural proteins. CEFs were infected with rMd5-HA-pLORF1, rMd5-HA-VP22, rMd5-HA-VP11/12, rMd5-HA-VP13/14, or rMd5-HA-VP19C for 3 days. The cells were fixed and stained with anti-HA antibody, followed by either Alexa 488 or Alexa 555-conjugated goat anti-rabbit IgG. Subsequently, the cells were treated with anti-pLORF1 antibody and either Alexa 555 or Alexa 488-conjugated goat anti-mouse IgG. Zoomed-in views of representative cells, indicated by white boxes, are shown in Fig 7D.
(TIF)

S1 Data Excel containing the numerical data underlying Fig 2. (XLSX)

S2 Data Excel containing the numerical data underlying Fig 3. (XLSX)

S3 Data Excel containing the numerical data underlying Fig 4. (XLSX)

S4 Data Excel containing the numerical data underlying S2 Fig. (XLSX)

S5 Data Excel containing the numerical data underlying S5 Fig. (XLSX)
