# Supplementary material for: Marek’s disease virus-1 unique gene LORF1 is involved in viral replication and MDV-1/Md5-induced atrophy of the bursa of Fabricius
Source: PLoS Pathog. 2025 Feb 3;21(2):e1012891. doi: 10.1371/journal.ppat.1012891 (PMC11790089; doi:10.1371/journal.ppat.1012891)
Supplement: S1 Table — (DOCX) [file ppat.1012891.s001.docx]

S1 Table. Bursa/body weight ratios in experimental chickens at different time points post-inoculation

| **Bursa/body weight ratio (%)**  **(Bursa weight [g] / body weight [g])** | | | | | |
| --- | --- | --- | --- | --- | --- |
| Time | Control | rMd5 | rMd5  ΔLORF1 | rMd5  -reLORF1 | rMd5  ΔMeq |
| 7 dpi | 0.37  (0.40/108) | 0.29  (0.35/122) | 0.41  (0.48/118) | 0.23  (0.19/84) | 0.38  (0.43/114) |
|  |  |  | 0.31  (0.31/100) | 0.23  (0.21/92) | 0.31  (0.27/88) |
| 14 dpi | 0.43  (0.88/203) | 0.23  (0.30/129) | 0.39  (0.70/180) | 0.26  (0.43/166) | 0.20  (0.33/167) |
|  |  |  | 0.61  (1.04/171) | 0.24  (0.34/144) | 0.45  (0.85/187) |
| 21 dpi | 0.58  (1.98/341) | 0.18  (0.32/177) | 0.42  (1.38/239) | 0.09  (0.12/128) | 0.06  (0.15/258) |
|  |  |  | 0.34  (0.88/262) | 0.23  (0.59/252) | 0.09  (0.17/190) |
| 28 dpi | 0.54  (2.48/458) | 0.07  (0.17/239) | 0.42  (1.40/331) | 0.23  (0.72/308) | 0.14  (0.46/318) |
|  |  |  | 0.29  (0.94/324) | 0.11  (0.24/221) | 0.12  (0.36/301) |
| 35 dpi | 0.47  (2.31/489) | 0.12  (0.48/385) | 0.55  (3.43/625) | 0.05  (0.19/355) | 0.06  (0.33/485) |
|  |  |  | 0.42  (2.58/610) | 0.08  (0.28/345) | 0.10  (0.44/440) |
| 42 dpi | 0.43  (2.52/590) | 0.06  (0.19/345) | 0.22  (0.55/245) | 0.10  (0.42/405) | 0.06  (0.32/545) |
|  |  |  | 0.16  (0.61/375) | 0.08  (0.19/244) | 0.10  (0.52/510) |
