# Supplementary material for: Marek’s disease virus-1 unique gene LORF1 is involved in viral replication and MDV-1/Md5-induced atrophy of the bursa of Fabricius
Source: PLoS Pathog. 2025 Feb 3;21(2):e1012891. doi: 10.1371/journal.ppat.1012891 (PMC11790089; doi:10.1371/journal.ppat.1012891)
Supplement: S2 Table — (DOCX) [file ppat.1012891.s002.docx]

S2 Table. Primers used for the construction of expression plasmids

| **Name** | **Sequence (5’→3’)** |
| --- | --- |
| His-pLORF1-F | AATGGATCCATGTCTTGCACGCGGGGACGAGCAAA |
| His-pLORF1-R | AATGCGGCCGCTTGGTTCGCAGTGC GAACGC |
| GFP-pLORF1-F | GCAGTCGACATGGTGAATAGACGCAACTATAATTACTC |
| GFP-pLORF1-R | GGTGGATCCTTATTGGTTCGCAGTGCGAACG |
| GFP-VP22-F | GATCTCGAGCTATGGGGGATTCTGAAAGGCG |
| GFP-VP22-R | GGTGGATCCTTATTCGCTATCACTGCTACGATATCCG |
| GFP-VP11/12-F | GATCTCGAGCTATGAAGCGGCTCAGCTCTTCTG |
| GFP-VP11/12-R | GCAGAATTCTCAATCGGTAGCCACCCTCAACC |
| GFP-pUL11-F | GATCTCGAGCTATGGGCCAAGCAGTGTCG |
| GFP-pUL11-R | GCAGAATTCTCATTCTTTATTAAACATCATAACATACTTTTCATGATC |
| GFP-pUL51-F | GATCTCGAGATGCAAACCAGCTCAAGAACATACG |
| GFP-pUL51-R | GCAGAATTCTTATAATTCGGTAATGAGATTATTTTCCC |
| Flag-pLORF1-F | CCAGGATCCATGGTGAATAGACGCAACTATAATTACTCAATG |
| Flag-pLORF1-R | CGTTCGCACTGCGAACCAATAAGCGGCCGCGTC |
| Flag-VP22-F | GCTGGATCCCCGGAATTCATGGGGGATTCTGAAAGGCG |
| Flag-VP22-R | ATGCATGCTCGAGTTATTCGCTATCACTGCTACGATATCCG |
| Flag-VP11/12-F | CCGGAATTCATGAAGCGGCTCAGCTCTTC |
| Flag-VP11/12-R | ATGCTCGAGTCAATCGGTAGCCACCCTCAACC |
| Flag-pUL11-F | CCGGAATTCATGGGCCAAGCAGTGTCGTA |
| Flag-pUL11-R | ATGCTCGAGTCATTCTTTATTAAACATCATAACATACTTTTCATGATC |
| Flag-pUL51-F | CCGGAATTCATGCAAACCAGCTCAAGAACATAC |
| Flag-pUL51-R | ATGCTCGAGTTATAATTCGGTAATGAGATTATTTTCCCTAGAAAAC |
| HA-VP22-F | CCGGAATTCATGGGGGATTCTGAAAGGCG |
| HA-VP22-R | ATGCTCGAGTTATTCGCTATCACTGCTACGATATCCG |
| HA-VP11/12-F | CCGGAATTCATGAAGCGGCTCAGCTCTTC |
| HA-VP11/12-R | ATGCTCGAGTCAATCGGTAGCCACCCTCAACC |
| HA-VP13/14-F | CTGGCGGCCGCGTATGCAAATGCCTTCTATGCATC |
| HA-VP13/14-R | ATAGGGCCCTCAATTCGCCCGTTGTGG |
| HA-VP19C-F | CCGGAATTCATGAAACCACTCTTACGATCGC |
| HA-VP19C-R | ATGCTCGAGTTAATAACATTCGATCCATGTACCTATATTCC |
| HSV1-VP22-F | GTTCCAGATTACGCTGAATTCATGACCTCTCGCCGCTCCG |
| HSV1-VP22-R | ATTAAGATCTGCTAGCTCGAGTCACTCGACGGGCCGTCTGGG |
| HSV1-VP11/12-F | GTTCCAGATTACGCTGAATTCATGCAGCGCCGGACGCGCGG |
| HSV1-VP11/12-R | ATTAAGATCTGCTAGCTCGAGTCACCGGCTCCGGCGTCCTTCGCG |
| HSV1-VP13/14-F | GTTCCAGATTACGCTGAATTCATGTCGGCTCGCGAACCCGC |
| HSV1-VP13/14-R | ATTAAGATCTGCTAGCTCGAGTTATGGGCGTGGCGGGCCTCCC |
| HSV1-VP19C-F | GTTCCAGATTACGCTGAATTCATGAAGACCAATCCGCTACCCGC |
| HSV1-VP19C-R | ATTAAGATCTGCTAGCTCGAGTCACGCGCATGCCCGCCACTCG |
