# Supplementary figures and images for: Marek’s disease virus-1 unique gene LORF1 is involved in viral replication and MDV-1/Md5-induced atrophy of the bursa of Fabricius
Source: PLoS Pathog. 2025 Feb 3;21(2):e1012891. doi: 10.1371/journal.ppat.1012891 (PMC11790089; doi:10.1371/journal.ppat.1012891)

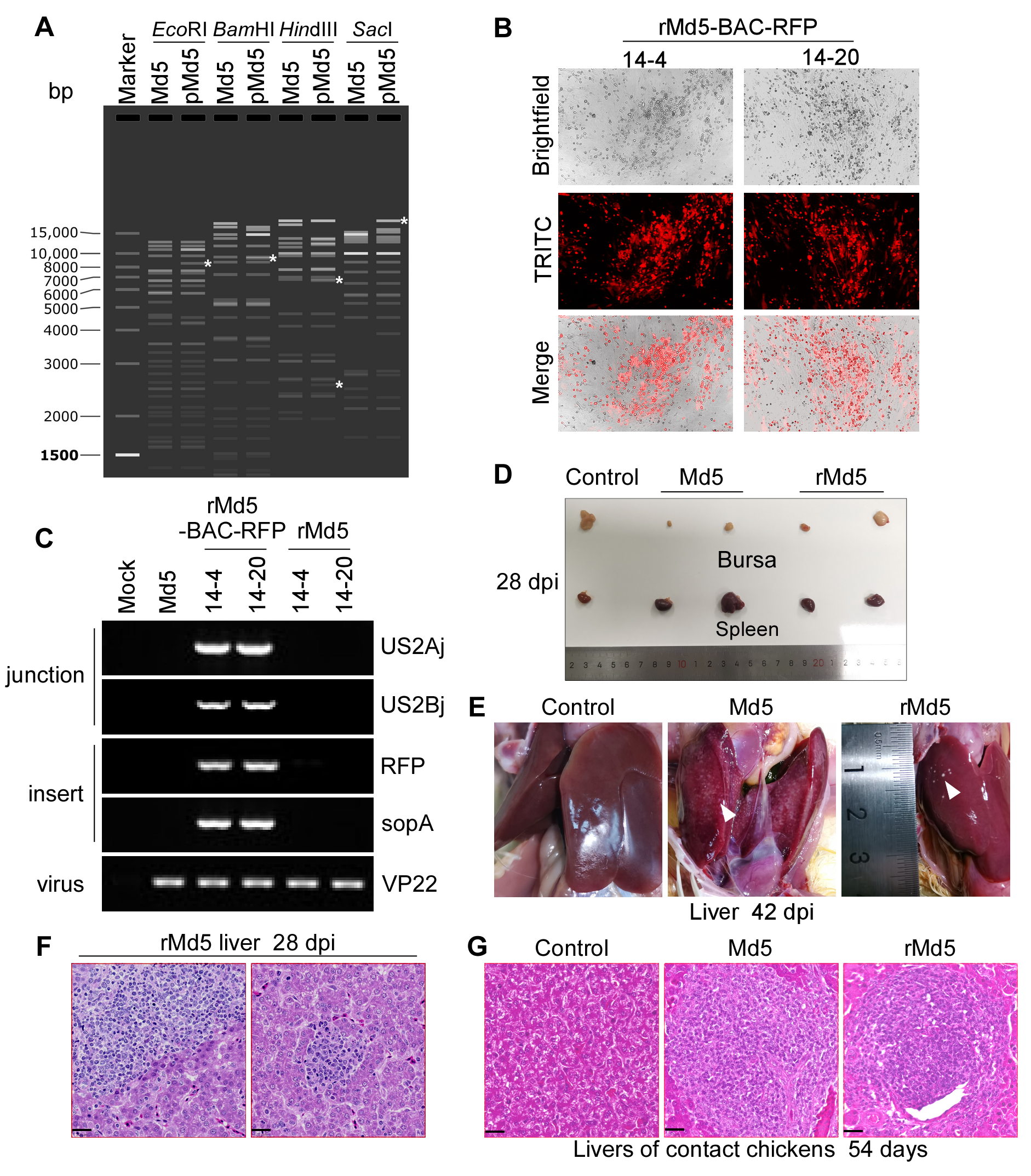

Supplement: S1 Fig — (A) The image shows a simulated experiment using the SnapGene software. Md5 or pMd5 BAC genomes are digested with EcoRI, BamHI, HindIII, or SacI on a 0.8% agarose gel. The bands marked with asterisks indicate the insertion of the transfer vector. (B) Recombinant viruses rMd5-BAC-RFP clones 14–4 and 14–20 were generated by transfecting CEFs with pMd5 DNA. At 5 days post-transfection, plaques expressing RFP were observed under fluorescence microscopy. (C) Recombinant viruses rMd5/14-4 and rMd5/14-20 were obtained by co-transfecting CEFs with pMd5 and Cre expression plasmid. The junction fragments (US2Aj and US2Bj), inserted genes (RFP and sopA), and viral genes (VP22) in mock-, Md5-, rMd5-BAC-RFP, and rMd5-infected CEFs at 3 dpi were amplified by PCR. (D-G) The animal experiment was performed as described in “Materials and Methods.” (D) Images of the bursas and spleens from chickens of the Md5 or rMd5 group at 28 dpi. (E) Images of the livers from chickens in each group at 42 dpi. White arrows indicate tumor nodules in the livers. (F) Liver tissues infected with rMd5 at 28 dpi were stained with H&E. (G) Liver tissues from contact chickens at 54 dpi in each group were stained with H&E. Scale bar: 20 μm (F and G). (TIF) [file ppat.1012891.s003.tif]

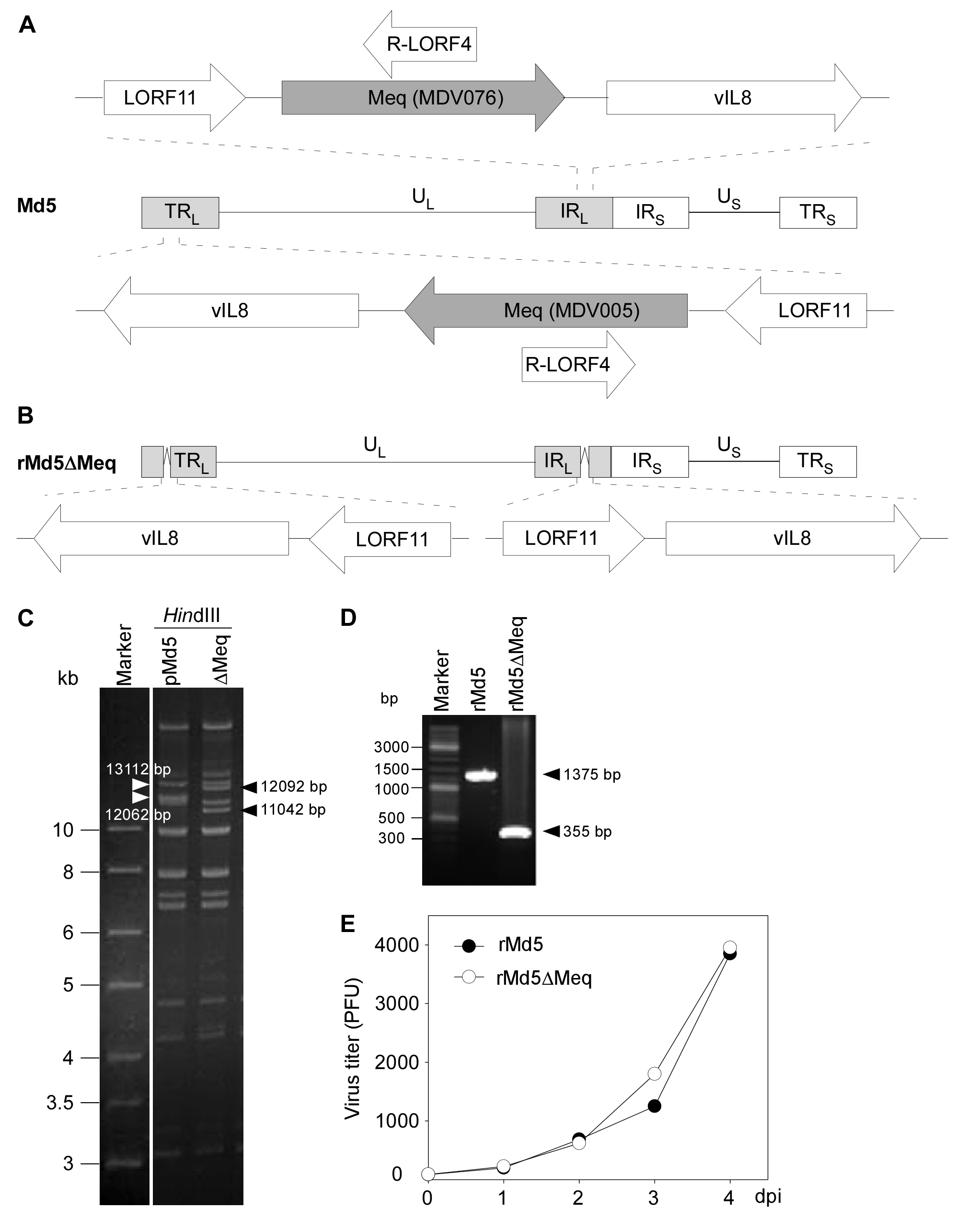

Supplement: S2 Fig — (A) The linear MDV-1 Md5 genome and a portion of the repeat regions (TRL and IRL) encoding genes LORF11 to vIL8 are depicted. (B) Schematic diagrams of the rMd5ΔMeq virus with a full-length deletion of the Meq gene. (C) DNA analysis of pMd5 and pMd5ΔMeq genomes. DNAs purified from BACs containing E. coli were digested with HindIII and separated on a 0.8% agarose gel. The bands that appeared after the deletion of the Meq gene are marked with arrows. (D) PCR amplification result of the Meq locus in pMd5 or pMd5ΔMeq. (E) CEFs infected with 100 PFU of rMd5 or rMd5ΔMeq were harvested at the indicated time points and subsequently seeded onto fresh CEFs. Viral plaques were counted at 7 dpi. Each value represents the mean of two independent duplicates. (TIF) [file ppat.1012891.s004.tif]

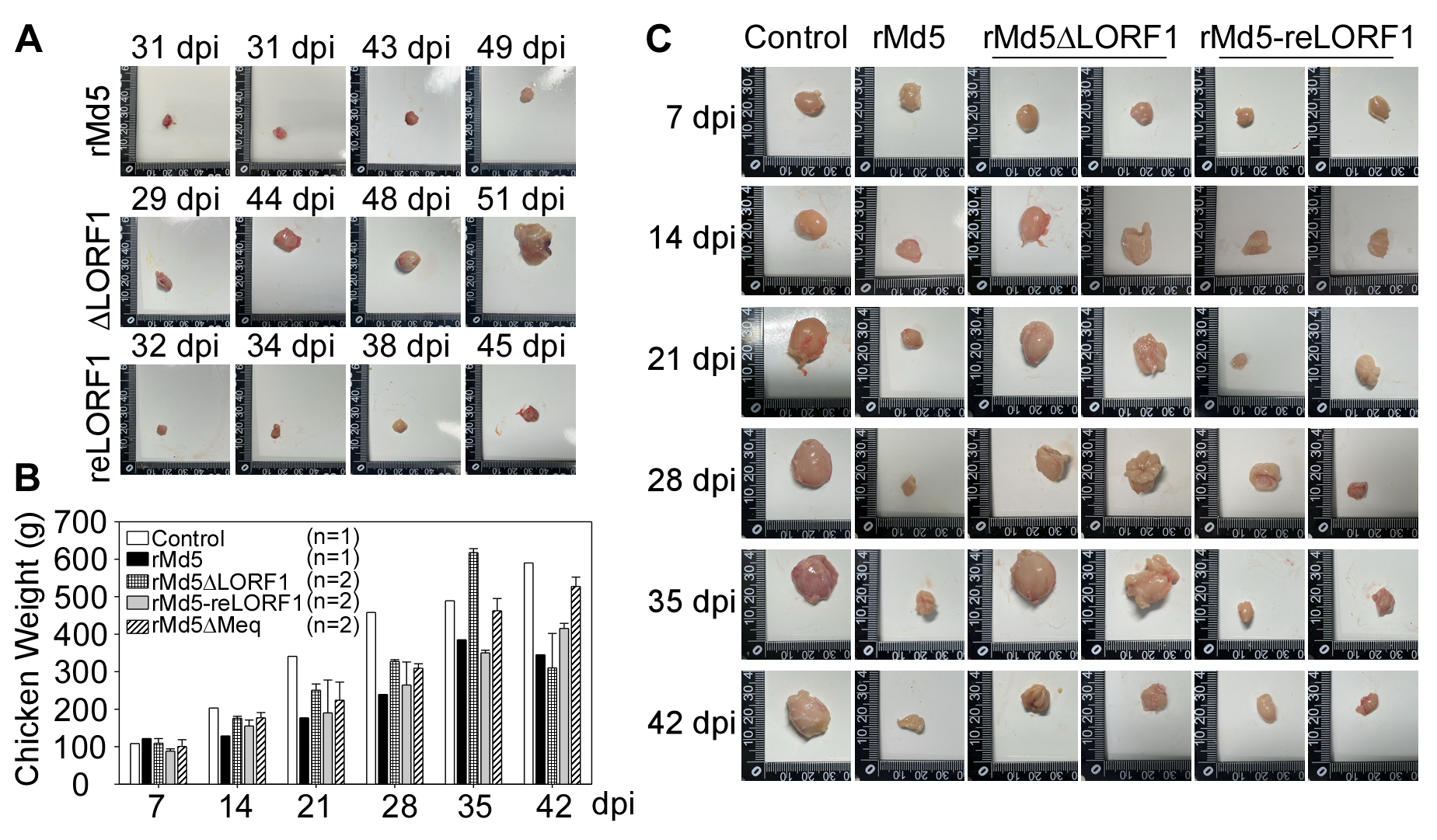

Supplement: S3 Fig — One-day-old SPF chickens were intraperitoneally inoculated with 1,000 PFU of rMd5, rMd5ΔLORF1, or rMd5-reLORF1, and DMEM as a negative control. The chickens were kept in isolators for daily monitoring of MD symptoms or death. (A) Images of bursas of four representative dead chickens from each group at the indicated time points. (B) The histogram of the body weights of chickens in each group at 7, 14, 21, 28, 35, and 42 dpi. Results represent the mean value, with error bars indicating the standard error of the mean. (C) Images of chicken bursas from each group at the indicated time points, as in B. (TIF) [file ppat.1012891.s005.tif]

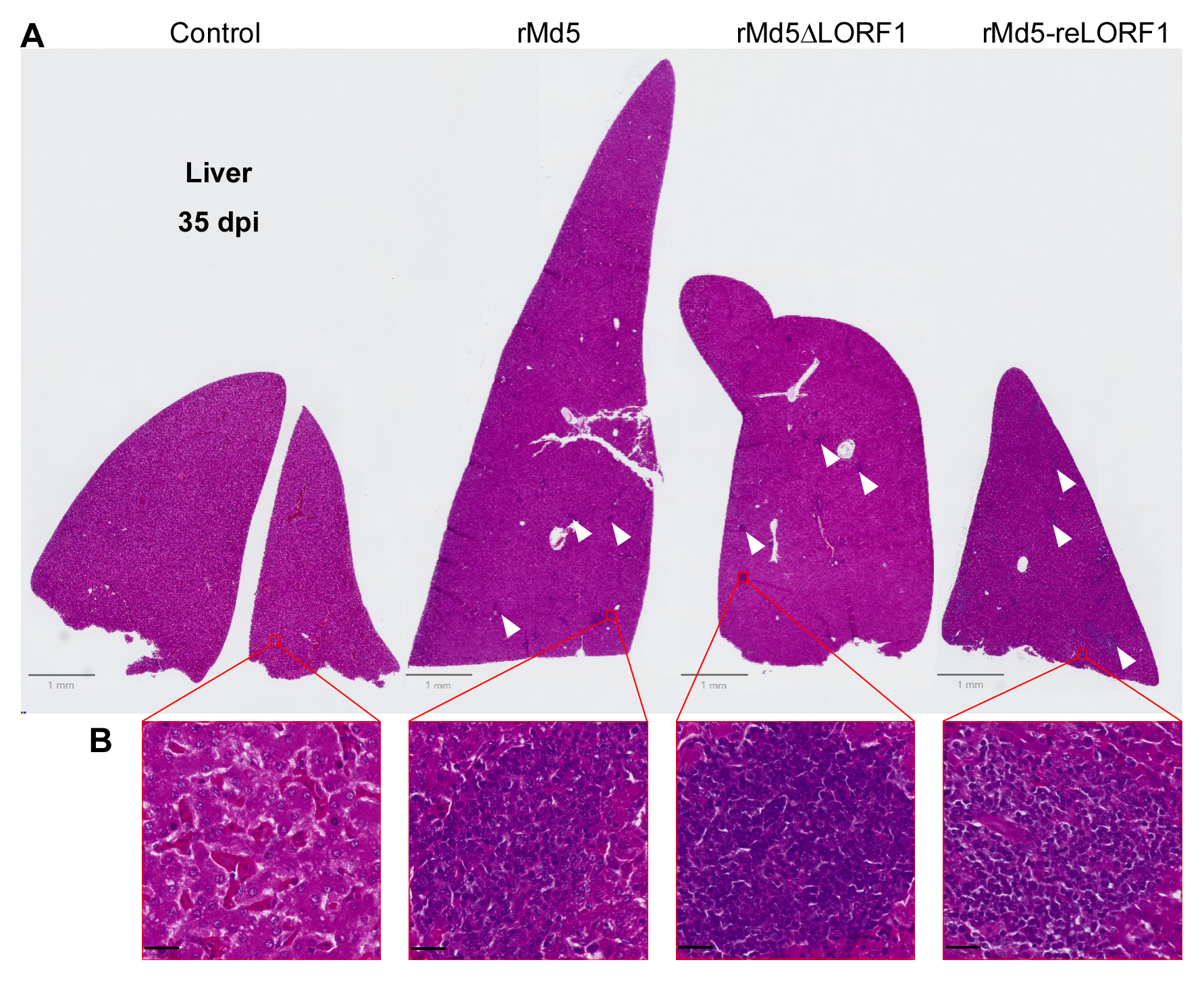

Supplement: S4 Fig — The liver tissues from chickens were mock-treated or inoculated with 1,000 PFU of rMd5, rMd5ΔLORF1, or rMd5-reLORF1 and euthanized at 35 dpi. (A) Focal infiltration of lymphoid tumor cells in the liver tissues is highlighted by white arrows (H&E). (B) A higher magnification of areas indicated by red boxes in A. Scale bar: 1 mm (A), 20 μm (B). (TIF) [file ppat.1012891.s006.tif]

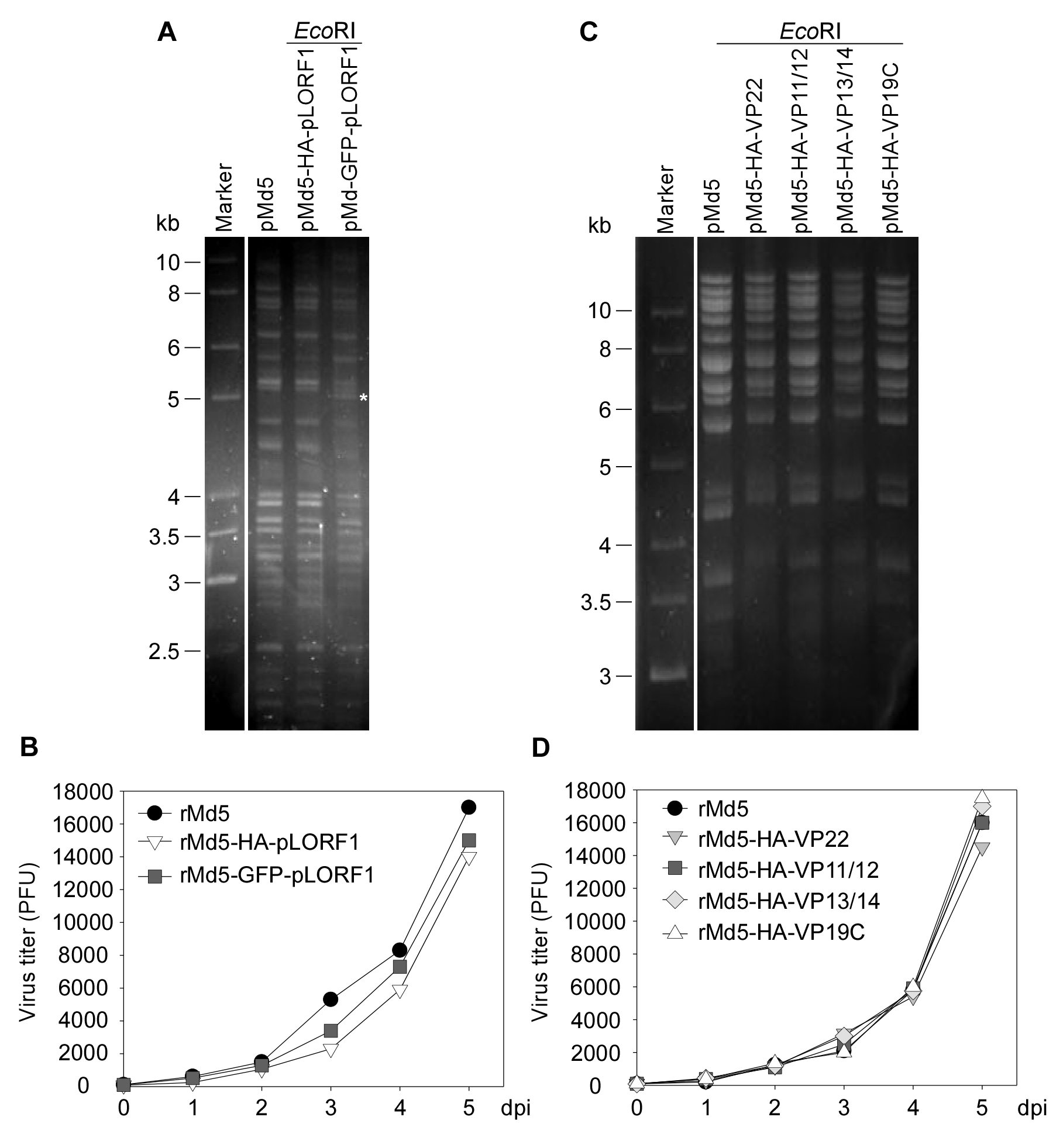

Supplement: S5 Fig — (A and C) RFLP analysis of HindIII-digested BAC DNAs, including pMd5, pMd5-HA-pLORF1, pMd5-GFP-pLORF1, pMd5-HA-VP22, pMd5-HA-VP11/12, pMd5-HA-VP13/14, and pMd5-HA-VP19C. (B and D) The growth curve of recombinant viruses, including rMd5, rMd5-HA-pLORF1, rMd5-GFP-pLORF1, rMd5-HA-VP22, rMd5-HA-VP11/12, rMd5-HA-VP13/14, and rMd5-HA-VP19C. Each value represents the mean of two independent duplicates. (TIF) [file ppat.1012891.s007.tif]

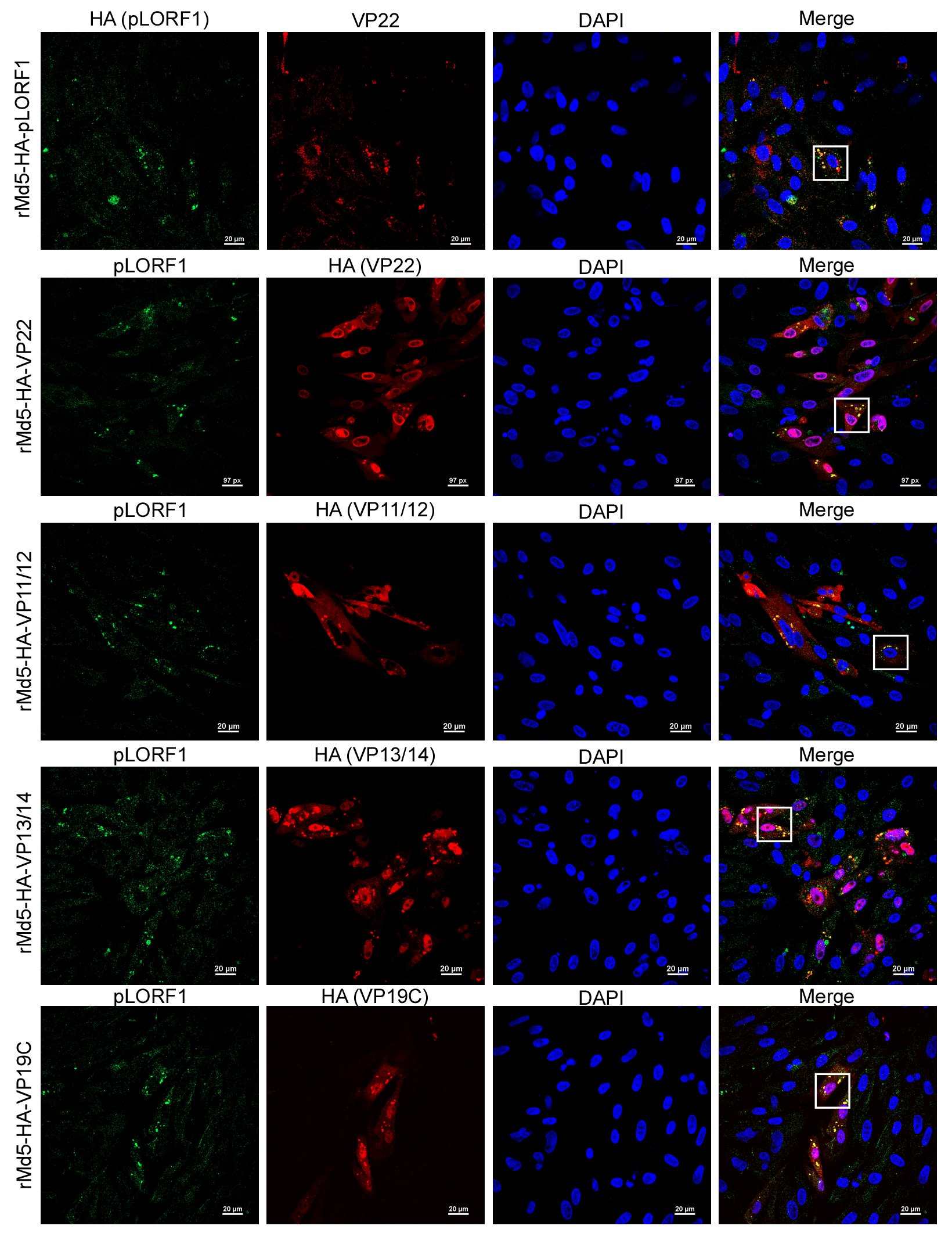

Supplement: S6 Fig — CEFs were infected with rMd5-HA-pLORF1, rMd5-HA-VP22, rMd5-HA-VP11/12, rMd5-HA-VP13/14, or rMd5-HA-VP19C for 3 days. The cells were fixed and stained with anti-HA antibody, followed by either Alexa 488 or Alexa 555-conjugated goat anti-rabbit IgG. Subsequently, the cells were treated with anti-pLORF1 antibody and either Alexa 555 or Alexa 488-conjugated goat anti-mouse IgG. Zoomed-in views of representative cells, indicated by white boxes, are shown in Fig 7D. (TIF) [file ppat.1012891.s008.tif]
